# Microbial model communities exhibit widespread metabolic interdependencies

**DOI:** 10.1038/s42003-025-09306-y

**Published:** 2025-12-03

**Authors:** Armando Pacheco-Valenciana, Anna Tausch, Iva Veseli, Jennah E. Dharamshi, Fabian Bergland, Luis F. Delgado, Alejandro Rodríguez-Gijón, Anders F. Andersson, Sarahi L. Garcia

**Affiliations:** 1 Department of Ecology, Environment, and Plant Sciences, Science for Life Laboratory, Stockholm University, Stockholm, Sweden; 2 Institute for Chemistry and Biology of the Marine Environment (ICBM), School of Mathematics and Science, Carl von Ossietzky Universität Oldenburg, Oldenburg, Germany; 3 Helmholtz Institute for Functional Marine Biodiversity at the University of Oldenburg (HIFMB), Oldenburg, Germany; 4 Alfred Wegener Institute, Helmholtz Centre for Polar and Marine Research, Bremerhaven, Germany; 5 Department of Gene Technology, Science for Life Laboratory, KTH Royal Institute of Technology, Stockholm, Sweden

**Keywords:** Metagenomics, Water microbiology, Microbial ecology

## Abstract

Microorganisms thrive in complex communities shaped by intricate interactions, yet the extent and ecological implications of biosynthetic dependencies in natural communities remain underexplored. Here, we used a dilution approach to cultivate 204 microbial model communities from the Baltic Sea and recovered 527 metagenome-assembled genomes (MAGs) that dereplicated into 72 species-clusters (>95% average nucleotide identity, ANI). Of these species, at least 70% represent previously uncultivated lineages. Combined with 1073 MAGs from Baltic Sea metagenomes, we generated a genomic catalog of 701 species-clusters. Our results show that cultures with more than three species included microorganisms with smaller genome sizes, lower biosynthetic potential for amino acids and B vitamins, and higher prevalence and abundance in the environment. Moreover, the taxa found together in the same model communities had complementary biosynthetic gene repertoires. Our results demonstrate that cultivating bacteria in dilution model communities facilitates access to previously uncultivated but abundant species that likely depend on metabolic partners for survival. Together, our findings highlight the value of community-based cultivation for unraveling ecological strategies. Finally, we confirm that metabolic interdependencies and genome streamlining are widespread features of successful environmental microorganisms.

## Abstract (web summary)

Microbial model communities bridge the gap between pure culture and environmental studies, revealing widespread metabolic interdependencies that shape how natural microbial groups thrive and interact with each other, emphasizing the ecological value of cultivating communities rather than individuals.

## Introduction

Microbial communities in diverse environments operate as complex systems driven by multi-species interactions^1^. Understanding such complex interactions is essential because microorganisms play key roles in the biogeochemical cycles on Earth^2^. To unravel microbial interactions, we need to investigate microbial communities at various levels of biological organization^3,4^, ranging from one-to-one species interactions to simplified multi-species systems (e.g., model communities^5^, synthetic communities^6^, or microcosms^7^), and ultimately to naturally occurring communities.

At the community level, metagenomics has become a powerful tool for uncovering the genetic potential of microbial communities via shotgun sequencing^8–10^. Analyzing metagenomic data reveals not only the vast diversity of microbial species^11^ but also their metabolic potential and co-occurrence networks, which are important for understanding ecosystem functioning^12^. To bridge the gap between broad metagenomic insights and detailed ecological understanding, a few studies have explored genome-specific traits and potential interactions by inferring auxotrophies. While auxotrophs have historically been experimentally identified via cultures that require the addition of specific nutrients to grow^13^, recent work based on genomes and metagenomes has determined auxotrophies based on pathway completeness and found smaller genomes to be more auxotrophic^6,14–19^. In all these studies, auxotrophy has been treated as a binary trait, however, microbial biosynthetic capabilities in nature likely span a spectrum. For example, many microorganisms can complete the biosynthesis of an essential metabolite starting from a precursor or intermediate without needing the essential metabolite itself^20,21^. Nevertheless, modeling work has shown that microbial communities enriched in the so-called auxotrophs can exhibit greater robustness under ecological disturbances, suggesting that these metabolic interdependencies may contribute to overall community stability^22^. While metagenomics offers a broad understanding of microbial communities, interactions cannot easily be inferred from co-occurrences within natural complex ecosystems.

Experimental systems are needed to observe microbial interaction dynamics under controlled conditions. Studies have increasingly turned to simplified systems^23–25^, such as co-cultures^26–28^ and mixed cultures^18,29,30^ to identify specific types of interactions. To further contextualize these findings, our literature review in Supplementary Data 1 provides a comprehensive overview of publications where microbial interaction patterns were observed in different experimental settings. Across these studies, cross-feeding mechanisms and mutualistic interactions are mostly studied in cultures with two different populations or species^27,31,32^. To a lesser extent, more complex metabolic interactions have also been studied by mixing different isolated species or co-cultivating them in model ecosystems, with the goal of increasing complexity to more closely resemble natural environments^29,33^. However, many of these methods focus on cultured isolates, and the vast majority of microorganisms remain uncultivated^34^. An alternative, yet underutilized method for establishing model ecosystems composed of previously uncultivated microorganisms is through dilution cultivation. Dilution to mixed cultures of naturally co-occurring microorganisms has the potential to cultivate previously uncultivated microorganisms as well as to allow observation of natural microbial interactions^35^. Such cultures, also known as microbial model communities, represent a small subset of the many interactions likely occurring in the natural systems^36^. By studying a larger number of microbial model communities, we can gain a more comprehensive understanding of microbial interactions occurring in natural environments.

In our study, we focused on studying potential interdependencies at two levels of biological organization by using high-throughput dilution cultivation of model communities together with genome-resolved metagenomics to unravel the ecological strategies of microorganisms in the Baltic Sea. Moreover, we examined the biosynthesis of essential metabolites or anabolic independence as a continuous spectrum rather than through conventional binary classifications. For this, we used pathway completeness metrics rather than assigning genomes as strictly prototrophic or auxotrophic. Finally, we identified correlations between genome size, potential biosynthesis of essential metabolites, relative abundance, and prevalence using genomes obtained from both microbial model communities and metagenomic data from Baltic Sea pelagic samples. Our findings demonstrate that microbial model communities are an effective technique for cultivating previously uncultivated taxa and for identifying putative microbial interactions, including metabolic interdependencies between biosynthetically dependent members.

## Results

### A Baltic Sea MAG catalog

**Fig. 1:**
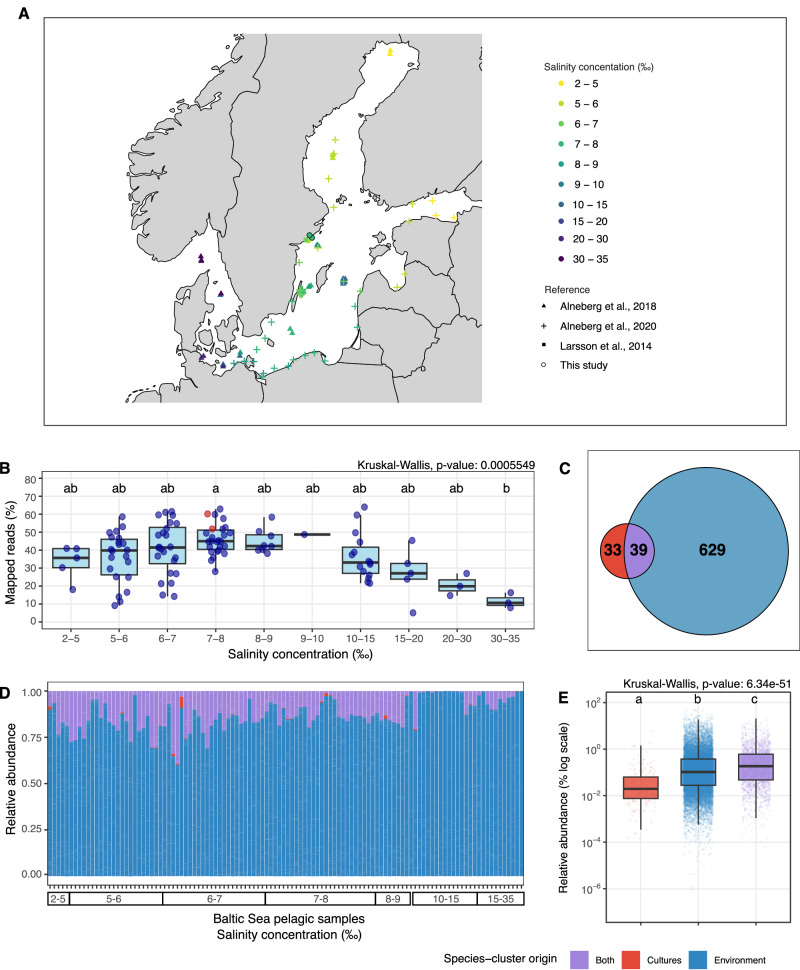
The BalticMAG catalog. **A** Map of the Baltic Sea showing the geographic location of all the metagenomes analyzed in this study, including our sampling site (*n* = 112). Sampling sites are color-coded according to their salinity gradient, measured in ‰ (parts per thousand, equivalent to PSU). The shapes correspond to the different reference sources. **B** Boxplot showing the percentage of mapped reads from all metagenomes to our species-cluster collection, categorized by salinity concentration (Kruskal–Wallis test). **C** Venn diagram showing the overlap of species-clusters presence among cultures (red), the environment (blue), and found in both (purple). **D** Relative abundance of species-clusters across the collection of metagenomes, ordered by salinity gradient from left to right (corresponding to the order in **B**). **E** Boxplot comparing the relative abundance of species-clusters categorized by their presence in cultures, the environment, or both (Kruskal–Wallis test). Kruskal–Wallis tests were followed by Dunn’s post hoc test for pairwise comparisons. Groups sharing at least one letter (e.g., a and ab) are not significantly different from each other, while groups with different letters (e.g., a vs. b) are significantly different (*p* < 0.05). Boxplots show the median and the interquartile range (IQR), with whiskers extending to 1.5× IQR. Publicly available Baltic Sea metagenomes were included in the analysis^37–39^.

To generate the microbial model communities, we used the dilution-to-extinction cultivation technique in two formats. The first type, low inoculum size, involved inoculating between approximately 2 and 100 cells per well in 1 mL 96-well plates used for each of the inoculum sizes. The second type, high inoculum size, ranged from approximately 200 to 1 × 10^6^ cells inoculated per microbial model community in 100 mL volumes. After a 4-week incubation period, we sent an aliquot of all 801 cultures for lysis and DNA amplification using multiple displacement amplification (MDA). Based on amplification success, 315 cultures passed the negative control threshold and were sent for sequencing. In total, only 204 microbial model communities together yielded 527 MAGs. Moreover, from the original sample used to establish the microbial model communities, we generated two metagenomes (each from a distinct DNA extraction method) that yielded 305 MAGs. To create a comprehensive genomic catalog, we also added 771 MAGs from 110 publicly available Baltic Sea metagenomes (Fig. 1A, Supplementary Data 2)^37–39^.

Examining this comprehensive MAG catalog allowed us to assess microbial genomic characteristics of all microorganisms found both in the environmental samples and our 204 microbial model communities (Supplementary Data 3). Altogether, the 1603 MAGs were dereplicated into 701 species-clusters (ANI > 95%), which form the Baltic Sea genomic catalog (BalticMAG catalog)^40^ used in this study (Supplementary Data 4). The average completeness of the 701 species-cluster representative MAGs is 88%, and they were all used to analyze taxonomy, abundance, prevalence, and estimated genome size (Supplementary Fig. 1). The varying completeness of the MAGs has a very minor effect on estimated genome size and relative abundance, as observed in other studies^17,41^. However, only 450 species-cluster representative MAGs are of high quality (completeness >90% and contamination <5%) and were used to investigate anabolic potential (Supplementary Fig. 1D).

Examining the source of genomes in the BalticMAG catalog, we found that 33 of the species-clusters included MAGs exclusively from the microbial model communities, 629 included MAGs exclusively from the environmental metagenomes, and 39 (54% of all cultured species) included MAGs from both sources (Fig. 1C, Supplementary Data 4, Supplementary Fig. 2).

To investigate the relative abundance of the BalticMAG catalog, we mapped all the environmental metagenomic reads against the genome catalog. We observed that salinity significantly co-varied with the proportion of metagenomic reads that mapped to all species-cluster representative genomes (Fig. 1B). While on average, 38.63% of the metagenomic reads per sample mapped to the BalticMAG catalog, the highest mapping percentages (63.99% and 62.89%) were observed at salinity concentrations of 11.28‰ and 7.65‰, respectively. Notably, the two metagenomes from this study (sample with salinity concentration of 7.12‰) displayed some of the highest mapping rates at 51.77% and 60.16%, reflecting that the BalticMAG catalog is most complete for salinities between 6 and 11‰.

Despite salinity differences (Fig. 1D), the average relative abundance of the 39 species-clusters that included MAGs from both microbial model communities and environmental metagenomes was significantly higher in the whole dataset (Fig. 1E), but also when comparing only the metagenomes from the location and salinity from which we sampled (Supplementary Fig. 3). This group of species-clusters (from both sources) shows that our cultivation method can capture some of the most abundant taxa from the environment. Altogether, the diverse taxa cultivated in model communities accounted for ~20% of the total relative abundance in the original environmental sample (Supplementary Fig. 4). Moreover, the 33 species-clusters with MAGs sourced exclusively from microbial model communities were detected across environmental metagenomes, albeit at significantly lower abundances. This indicates that our cultivation approach also enables the recovery of species that are missed by assembly and binning in metagenomic surveys.

### Higher inoculum size increases community richness and uncovers a genome size plateau

**Fig. 2:**
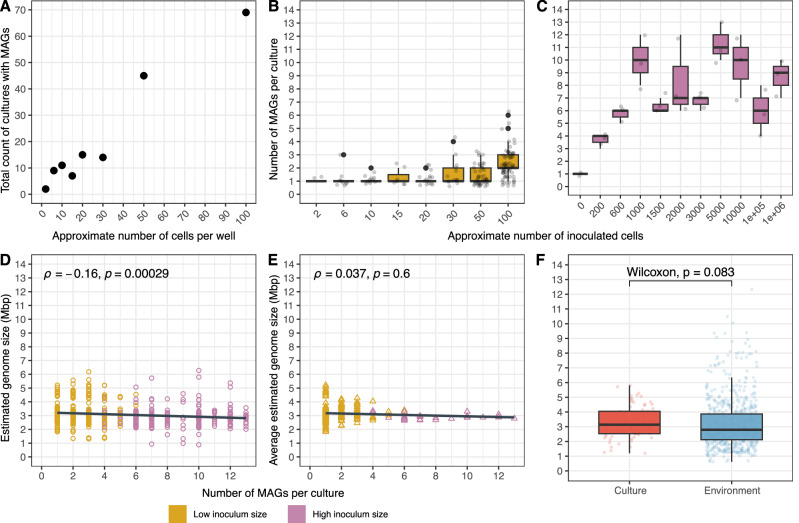
Impact of inoculum size on microbial richness and genome size. **A** Relationship between the number of inoculated cells (low inoculum size = 0–100 cells/well) and the total number of cultures from which MAGs were assembled and binned. **B** Number of MAGs per culture for the low inoculum size (yellow) and **C** the high inoculum size (pink) microbial model communities. **D** Estimated genome size (circles) of MAGs from cultures (*n* = 527) in relation to the number of species growing in the same culture, and **E** average estimated genome size (triangles) of MAGs growing in the same culture. **F** Boxplot comparing the average estimated genome size of species-clusters from cultures (*n* = 72) and those from the environment (*n* = 668). Statistical significance was assessed using the Wilcoxon rank-sum test. Boxplots show the median and the interquartile range (IQR), with whiskers indicating 1.5× IQR.

In the small inoculum size microbial model communities, the more cells we inoculated, the higher the number of cultures that yielded MAGs (Fig. 2A). In total, 94 low inoculum size model communities yielded only one MAG each, while 110 model communities resulted in two or more MAGs. In model communities with more than one MAG, each MAG belonged to a different species-cluster in our analysis. Therefore, for clarity, we refer to different MAGs within a model community as different species. Starting from an inoculum size of approximately 30 cells, microbial model communities with more than two species appear more often (Fig. 2B, C). Nearly 82% (*n* = 433) of the microbial model community MAGs were obtained from multi-species cultures, and the highest number of co-occurring species were found in the high inoculum size microbial model communities inoculated with 5000 cells (Fig. 2C). Despite using a complex inoculum from the Baltic Sea, observing a maximum of 13 co-occurring species suggests that our cultivation conditions may impose a threshold on the complexity of model communities. Alternatively, since sequencing followed MDA, there is also the possibility that some model communities included more species that were not amplified, assembled, or binned. Nevertheless, the increased growth success with increasing inoculum size likely reflects a greater probability of including cells that can grow in isolation or in the presence of a specific required community partner.

We found that as the number of retrieved MAGs per microbial model community increased, more microorganisms with smaller genomes emerged, as indicated by a slight but significant correlation between estimated genome size and the number of MAGs per microbial model community (Fig. 2D). In general, the estimated genome sizes ranged from 0.88 to 6.27 Mbp for MAGs from cultures and from 0.61 to 12.32 Mbp for MAGs from the environment (Fig. 2D, F). While there was no significant difference between the average estimated genome sizes of species-clusters from microbial model communities and Baltic Sea metagenomes (around 3.00 Mbp), we found that species-clusters exclusively composed of genomes from microbial model communities had significantly larger genome sizes than species-clusters including genomes from both sources, and no statistically different genome completeness or contamination (Supplementary Fig. 5). Further analysis reinforced the observation that genomes from high inoculum size microbial model communities and from model communities with more than 3 species had on average, smaller genome sizes (2.89 and 2.92 Mbp, respectively; Supplementary Figs. 6 and 7). Genomes from large inoculum size microbial model communities were also, on average, more prevalent and had higher average relative abundance in the investigated Baltic Sea metagenomes. Finally, the average genome size per microbial model community stabilized at around 3 Mbp in cultures containing more than three species (Fig. 2E).

### Model communities with more than three species reveal distinct microbial diversity

**Fig. 3:**
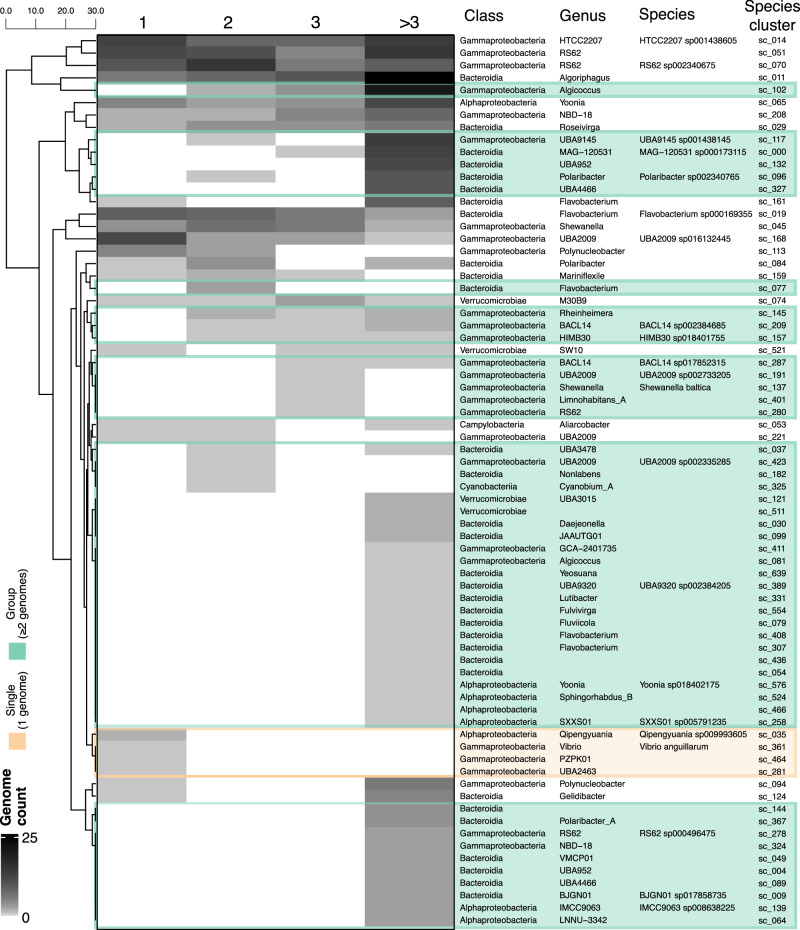
Microbial model communities of increased complexity host distinct sets of cultured species. Heatmap showing the distribution of the 72 species-clusters across cultures grouped by community complexity: 1, 2, 3, or more than 3 species per culture. Taxonomic affiliations (class, genus, and species) based on GTDB-Tk are displayed alongside their unique species-cluster IDs. The white-to-black gradient indicates the number of genomes recovered per species-cluster in each culture category. Species that exclusively grow alone are highlighted in a light orange box, while those only found to grow in groups (≥2 genomes) are highlighted in light green. Rows are clustered based on similarity in genome-count profiles, and the scale bar indicates the relative distance between clusters.

The 72 species from microbial model communities spanned five of the most abundant phyla (*Pseudomonadota*, *Bacteroidota*, *Campylobacterota*, *Cyanobacteriota*, and *Verrucomicrobiota*) present in the Baltic Sea environmental metagenomic sample^42,43^. These species varied substantially in their observed growth strategies. Four of them were only found in single-species cultures, 48 exclusively in multi-species cultures, and 20 appeared growing both alone and in groups (Fig. 3). Of the species growing consistently in groups, nine grew across different levels of community complexity (e.g., 2, 3, or more than three species per culture), and 39 species were restricted to a single type of community complexity. Specifically, 31 species were recovered from microbial model communities with more than three species. Notably, 70% of these cultured species lacked a species-level assignment in the GTDB taxonomy, suggesting they represent previously uncultivated lineages with no characterized MAGs.

**Fig. 4:**
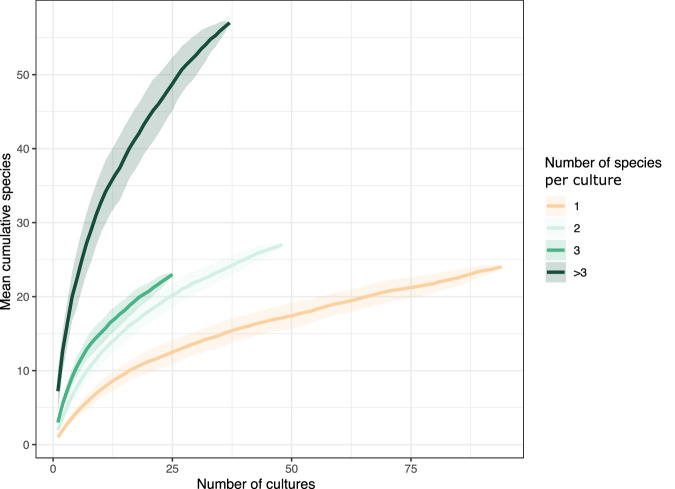
Culturing in groups enables access to a greater diversity of microbial species. Rarefaction curves show the cumulative number of unique species recovered from microbial model communities grouped by community complexity (1, 2, 3, or more than 3 species), as cultured samples are progressively added. For each category, species accumulation was calculated across 100 random permutations (bootstrap iterations), and shaded ribbons represent ±1 standard deviation from the mean. Communities with a single species (1 genome) are shown in light orange, while increasingly complex communities (≥2 genomes) are shaded in progressively darker greens.

To evaluate whether culturing bacteria in groups increases the cultivability and recovery of microbial diversity, we computed rarefaction curves for species accumulation across increasing numbers of cultures (Fig. 4). After 50 cultures of the single-species type, we had, on average, recovered 17 species, and every 12 new cultures would yield only three more species on average. As we cultivated more species together, the initial steep slope also increased. In fact, in 25 cultures with more than three species, 49 species were recovered. This demonstrates that culturing in groups is a powerful strategy for the recovery of microbial taxa that would normally not grow in axenic cultures.

### Species strictly found growing in groups are more abundant, prevalent, and have lower biosynthetic capacity

**Fig. 5:**
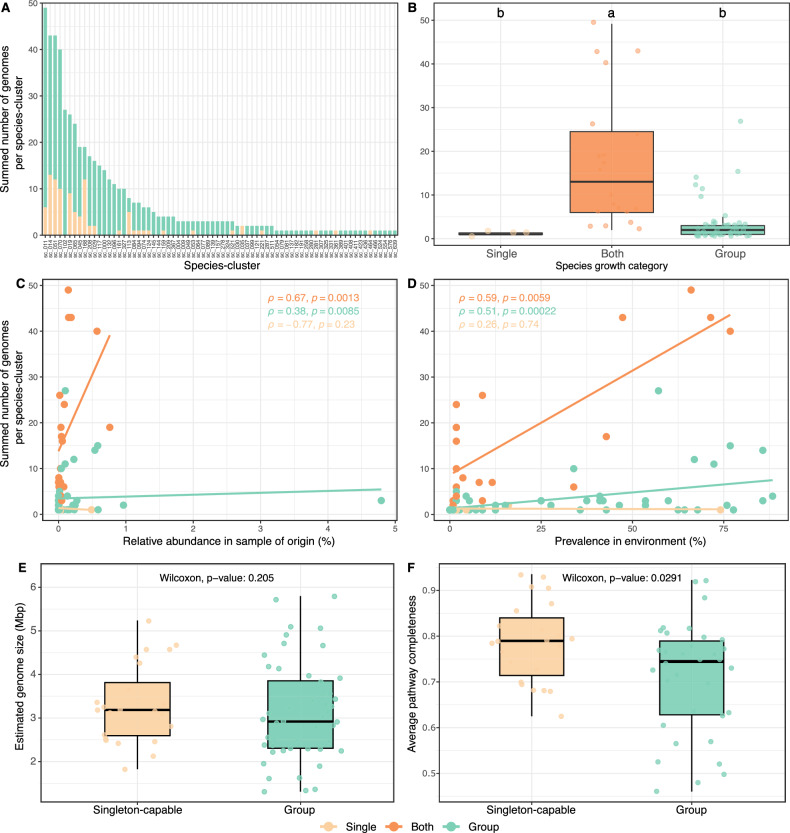
Growth strategy, environmental distribution, and genomic features of cultured microbial species. **A** Barplot showing the total number of cultured genomes recovered per species-cluster (*n* = 72). Bars are colored by the type of culture from which each genome was obtained: light orange for single-genome cultures and light green for multi-genome cultures. Species-clusters were further categorized based on their observed growth behavior: “Single” (light orange) for species that grew exclusively alone, “Group” (light green) for those that grew exclusively in groups, and “Both” (orange) for species that could grow alone and in groups. **B** Boxplot comparing the number of cultured genomes per species-cluster across the three growth categories. Kruskal–Wallis tests were followed by Dunn’s post hoc test for pairwise comparisons. Groups sharing at least one letter (e.g., a and ab) are not significantly different from each other, while groups with different letters (e.g., a vs. b) are significantly different (*p* < 0.05). **C** The dot plots display the relationship between the total number of cultured genomes per species-cluster and their relative abundance in the sample of origin (**D**), as well as the prevalence of these species-clusters in all environmental samples. Each data point represents one species-cluster. Associations were assessed using the Spearman’s rank correlation method (correlation coefficient *ρ* and *p* value shown). Trend lines are included in the plots for visualization purposes only. **E** Boxplot comparing the estimated genome size of species-cluster representative genomes (*n* = 72) growing exclusively in groups (“Group” = light green) and capable of growing alone (“Singleton-capable” = light orange). **F** Boxplot comparing the average pathway completeness of custom amino acid and vitamin biosynthesis modules between high-quality species-cluster representatives (*n* = 57; completeness >90%, contamination <5%) growing exclusively in groups and those capable of growing alone. Statistical significance was assessed using the Wilcoxon rank-sum test. Boxplots show the median and the interquartile range (IQR), with whiskers indicating 1.5× IQR.

The total number of genomes recovered per species-cluster varied considerably (Fig. 5A), ranging from those detected only once (e.g., sc_576 and sc_639; taxa of species-clusters can be found in Supplementary Data 4 and Fig. 3) to those found in over 40 model communities (e.g., sc_011 and sc_014). Fifteen species (21%) were present in more than 10 model communities, collectively accounting for approximately 63% of the total MAGs (330 out of 527). We found that most of the frequently retrieved species showed the flexibility of growing independently or in groups (Fig. 5A, B). However, our experiment did not systematically test if species found only on their own could also grow in groups, or if species found only in groups could also grow alone. Nevertheless, when we examined the relative abundance of all cultivated species across their source environmental sample, we observed a positive correlation with the total number of genomes recovered for species found in groups (Fig. 5C). On the other hand, the two most abundant species (Pelagibacterales sc_139 and Methylacidiphilales sc_121) were found exclusively in groups but were cultivated very few times. This might reflect that while many abundant microorganisms are easier to cultivate under our model community conditions, finding the right partners or getting the right conditions might be more challenging for others.

When examining species prevalence across all environmental samples, a similar significant positive correlation emerged with the number of cultured genomes per species (Fig. 5D), indicating that more prevalent taxa are more frequently retrieved through our cultivation conditions.

Next, we compared estimated genome sizes and biosynthetic potential for species capable of growing alone (singleton-capable) and those that strictly grow in groups. While there was no significant difference in estimated genome sizes between these two categories (Fig. 5E), we observed a significant difference in average pathway completeness of custom biosynthesis modules for both amino acids and vitamins, with species found strictly in groups exhibiting a lower average pathway completeness (Fig. 5F). These findings suggest that the ability to grow alone is most likely associated with greater anabolic independence, whereas species that require growing in groups may depend biosynthetically on other members of the community.

### Lowest anabolic independence and higher interdependencies in species growing in groups

**Fig. 6:**
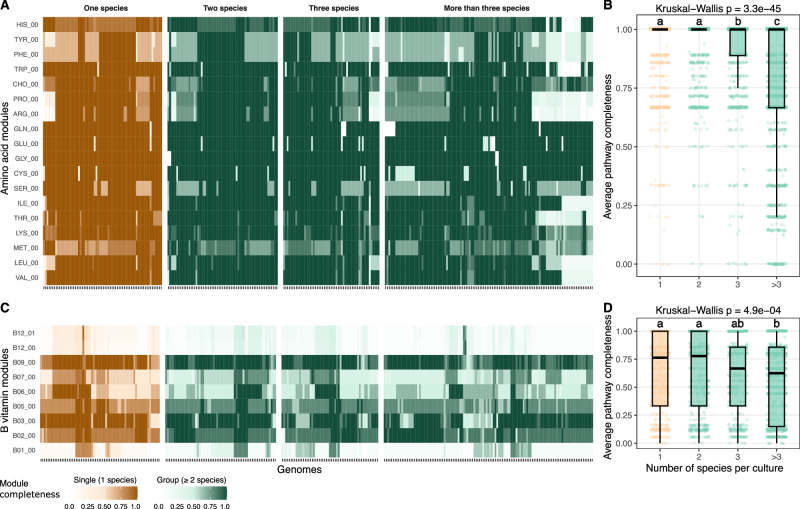
Amino acid and vitamin biosynthetic potential across species from varying community complexity. **A** Heatmap showing pathway completeness scores for 18 custom amino acid biosynthesis modules (three-letter abbreviations) across high-quality genomes (*n* = 305; >90% completeness, <5% contamination) grouped by community complexity: 1, 2, 3, or more than 3 species per culture. Each column represents a genome, and each row a biosynthetic module. Completeness values range from white (0) to dark orange (1) for genomes from single-genome cultures, and white to dark green for genomes from multi-genome cultures (≥2 genomes). **B** Boxplot summarizing the average amino acid pathway completeness per genome across the same four community complexity groups. **C** Heatmap as in (**A**), but for the 9 custom vitamin biosynthesis modules. Color gradients and grouping are defined identically. **D** Boxplot summarizing the average vitamin pathway completeness per genome across complexity groups. Statistical significance for (**B**, **D**) was assessed using Kruskal–Wallis tests, followed by Dunn’s post hoc test for pairwise comparisons. Groups sharing at least one letter (e.g., a and ab) are not significantly different from each other, while groups with different letters (e.g., a vs. b) are significantly different (*p* < 0.05). Boxplots show the median and the interquartile range (IQR), with whiskers indicating 1.5× IQR.

We next examined the biosynthetic potential of the 305 high-quality genomes (completeness >90%, contamination <5%) recovered from our microbial model communities, with a focus on how this potential relates to community complexity. We found that genomes from single-species and two-species cultures showed consistently higher anabolic independence in amino acid and vitamin module biosynthesis (Fig. 6 and Supplementary Fig. 8). In contrast, genomes from three-species cultures showed lower biosynthesis potential, and cultures with more than three species had the lowest anabolic independence. This reduction was particularly pronounced in amino acid biosynthesis pathways when compared to their highest average completeness value observed in cultures with one or two species. The nine amino acids with lowest pathway completeness were arginine (~25% lower), proline (~22%), phenylalanine (~21%), tyrosine (~20%), threonine (~18%), leucine (~17%), tryptophan (~16%), serine (~14%), and isoleucine (~12%) (Supplementary Fig. 9). Additionally, although vitamin B12 showed a relative decrease, both single-species and more than three-species model communities had low average pathway completeness (~22% down to ~12%), suggesting generally limited biosynthetic capacity for B12, regardless of community complexity.

While it is assumed that metagenome assembly might work better with lower diversity inputs, genomes from our more complex cultures (>3 species) showed only slight differences in completeness, contamination, and N50 values (Supplementary Fig. 10). To test whether this small difference in genome quality could explain the reduced biosynthetic potential in complex communities, we examined the relationship between genome completeness and the completeness of each biosynthetic module individually (Supplementary Fig. 11). Across all 27 modules, eight showed a significant positive correlation and four a significant negative correlation with an average *R*^2^ value of 0.03. Together, these results suggest that the lower biosynthetic capacity in multi-species cultures is a biological signal driven by community composition rather than a technical artifact of genome quality.

**Fig. 7:**
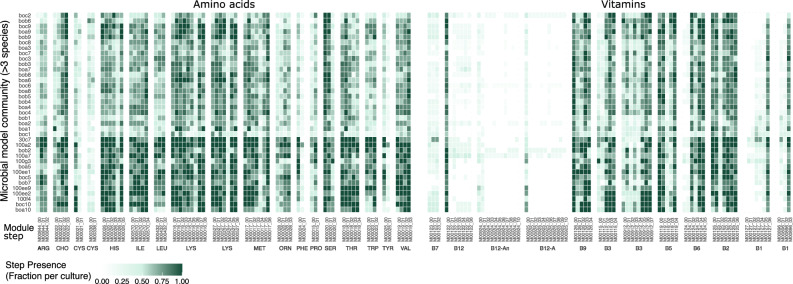
Community-level stepwise module framework analysis of amino acid and B vitamin biosynthetic pathways in complex model communities. Heatmap shows the average presence of biosynthetic module steps across 37 microbial model communities containing more than three species growing together, including all genomes regardless of genome completeness (*n* = 262). Each column represents a specific KEGG module step (e.g., M000020_01) grouped by compound. Each row corresponds to an individual model community. Values represent the proportion of genomes in each community that encode the respective module step, ranging from 0 to 1 (white to dark green). The stepwise framework uses canonical KEGG modules (non-customized), capturing the presence of standard and alternative steps within pathways.

To evaluate if microbial model communities with more than three species collectively encode complete biosynthetic pathways, we evaluated gene content (based on individual module steps) of all 262 genomes recovered from 37 microbial model communities, regardless of completeness. Even partial genomes were included because they can provide valuable evidence for individual metabolic steps. When examining the data at the community level, we observed that in these cultures, all species collectively encoded the biosynthetic pathways through a mosaic of partial contributions from different species (Fig. 7). This community-level stepwise completion indicates that biosynthetic capacity emerges collectively rather than within individual genomes, suggesting that anabolic interdependencies support the idea of facilitated community growth.

### Ubiquitous species tend to have smaller estimated genome sizes and reduced biosynthetic capacity

**Fig. 8:**
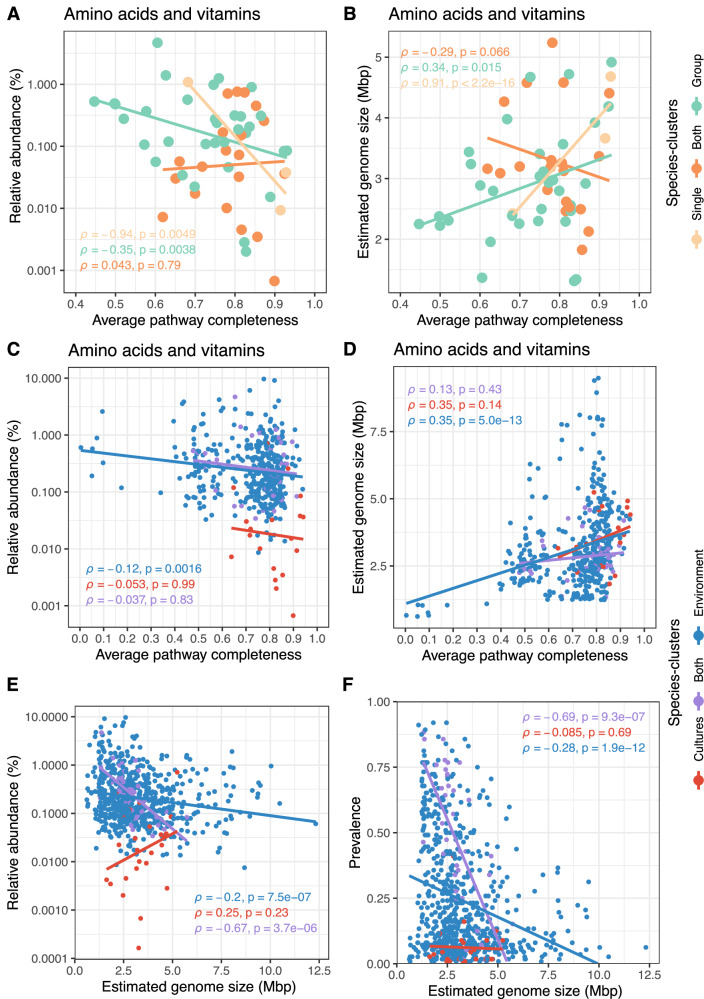
Link between pathway completeness, relative abundance, and estimated genome size of cultured species and the BalticMAG species catalog. **A** Correlation between average biosynthesis pathway completeness of custom amino acids and vitamin modules and the relative abundance of cultured species (*n* = 57, completeness >90%, contamination <5%). **B** Correlation between the average biosynthesis completeness for custom amino acid and vitamin modules and the estimated genome size of cultured species-clusters. Each data point represents one species cluster (*n* = 57, completeness >90%, contamination <5%), which either grew exclusively on their own (light orange), exclusively in groups (light green), or both on their own as well as in groups (orange). **C**, **D** replicate the analysis from (**A**, **B**) but include all species-clusters (*n* = 450, completeness >90%, contamination <5%) from both cultures and environmental samples. **E** Correlation between estimated genome size and average relative abundance and **F** correlation between estimated genome size and prevalence of all species-clusters (*n* = 701). Data points are color-coded to represent species-clusters from cultures only (red), environments only (blue), and those found in both (purple). All correlations use Spearman’s rank method, and the trend line is included only for visualization purposes. Publicly available metagenomes were included in the analysis^37–39^.

For high-quality species from model communities (*n* = 57), we found a significant negative correlation between relative abundance and average pathway completeness for both amino acids and vitamins in species that exclusively grew in groups and those that exclusively grew alone (Fig. 8A). This trend persisted for vitamin biosynthesis (Supplementary Fig. 12C), while amino acids alone showed a weaker association (Supplementary Fig. 12B). Moreover, we observed a significant positive correlation between estimated genome size and average pathway completeness for species that exclusively grew in groups and alone, showing that smaller genomes encode fewer biosynthetic pathways (Fig. 8B). Notably, the correlation between vitamin biosynthesis and estimated genome size was particularly strong in group-only species (Supplementary Fig. 12F).

Scaling the observations from model communities to all high-quality species from the BalticMAG catalog (*n* = 450), we found similar trends, particularly among species detected only in environmental metagenomes. In this group, we found that higher average relative abundances (Fig. 8C) and smaller estimated genome sizes (Fig. 8D) were linked to lower average completeness of biosynthetic pathways for amino acids and vitamins.

Finally, when we analyzed all 701 species-clusters, we observed a strong negative correlation between estimated genome size and both relative abundance (Fig. 8E) and prevalence across samples (Fig. 8F). These results align with prior observations, where the most successful and widespread taxa have a streamlined genome with low biosynthetic potential^17^. Collectively, these findings suggest that biosynthetic interdependencies may be a common ecological strategy in the Baltic Sea.

### Anabolic dependencies in the Baltic Sea: different paths to microbial success

**Fig. 9:**
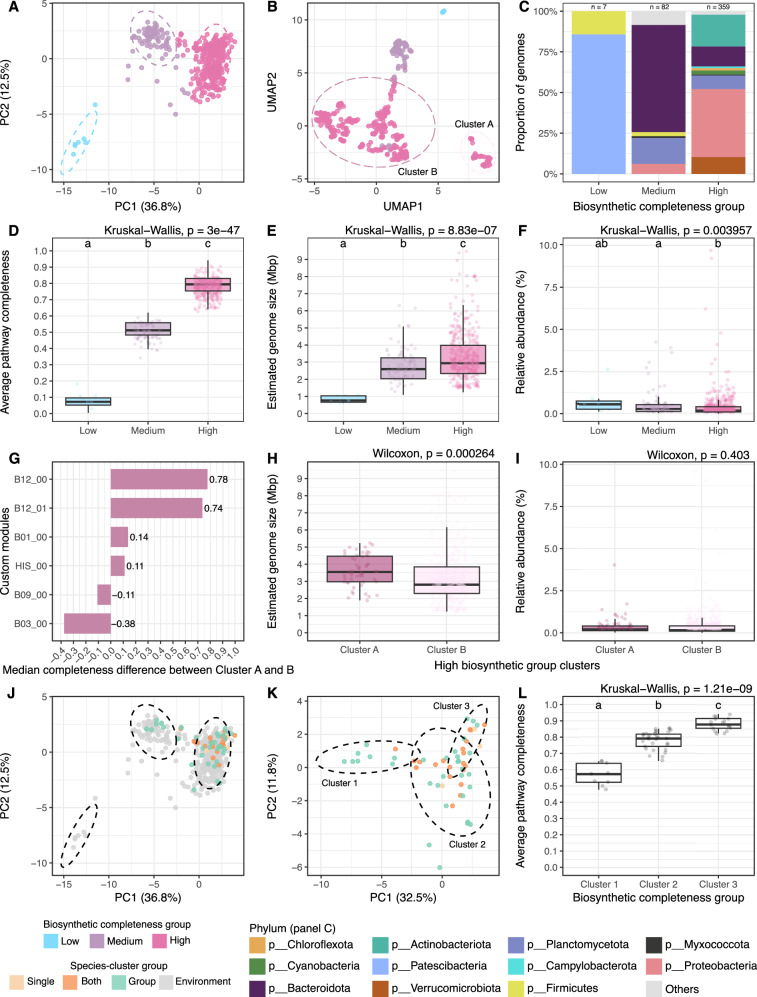
Anabolic dependencies and biosynthetic clustering of the BalticMAG species catalog, including cultivated species. **A** Principal component analysis (PCA) of the biosynthetic module completeness, with genomes color-coded by biosynthetic completeness group: Low = light blue, Medium = light purple, High = pink. **B** Uniform Manifold Approximation and Projection (UMAP) of the same data, confirming the biosynthetic groupings. **C** Stacked barplot showing the proportion and taxonomic composition of the three biosynthetic completeness groups, color-coded by phylum. Boxplots comparing **D** average pathway completeness, **E** estimated genome size (Mbp), and **F** relative abundance of species-clusters grouped by biosynthetic completeness. Statistical significance was assessed using a Kruskal–Wallis test (*p* < 0.05), followed by Dunn’s post hoc test with Bonferroni correction; groups with different letters are significantly different (*p* < 0.05). **G** Median completeness differences for KEGG custom biosynthesis modules between Cluster A and Cluster B genomes of the High biosynthetic group. Positive values indicate modules are more complete in Cluster A, while negative values indicate modules are more complete in Cluster B. Boxplots comparing **H** estimated genome size (Mbp) and **I** relative abundance of species-clusters from Cluster A (dark pink) and Cluster B (light pink). Statistical significance was assessed using the Wilcoxon rank-sum test (*p* < 0.05). **J** PCA of biosynthetic completeness color-coded by species-cluster growth category: Single = light orange, Group = light green, Both = orange; environmental-only clusters are shown in gray. **K** PCA of the 57 high-quality genomes from microbial model communities, also color-coded by growth category. **L** Boxplot showing the average pathway completeness of the three biosynthetic clusters derived from cultivated species. Statistical significance was tested using Kruskal–Wallis with Dunn’s post hoc test for pairwise comparisons. Groups sharing at least one letter (e.g., a and ab) are not significantly different from each other, while groups with different letters (e.g., a vs. b) are significantly different (*p* < 0.05). Boxplots show the median and the interquartile range (IQR), with whiskers indicating 1.5× IQR. Publicly available metagenomes were included in the analysis^37–39^.

Given that anabolic dependencies seem to be common in the Baltic Sea, an important question arises: Are these dependencies uniform across different microorganisms, or do different microorganisms adopt distinct metabolic strategies to achieve ecological success? To explore variation in biosynthetic potential across the BalticMAG catalog, we analyzed the genomes of 450 high-quality species. A principal component analysis (PCA) on the biosynthetic completeness matrix revealed a clear separation of genomes into three distinct biosynthetic completeness groups: Low (0–30%), Medium (30–62.5%), and High (>62.5%) (Fig. 9A, D). Estimated genome size followed the trend, increasing from low to high across the biosynthetic completeness groups (Fig. 9E). In general, among the most frequently incomplete or partially incomplete amino acid pathways were histidine, phenylalanine, and tyrosine as well as vitamin B1 and B12 (Supplementary Fig. 13). The microorganisms in the different biosynthetic groups showed distinct and clear taxonomic signatures (Fig. 9C). The low biosynthesis completeness group consisted exclusively of *Patescibacteria* and a single *Firmicutes* genome, both lineages associated with symbiotic or highly host-dependent lifestyles. The medium biosynthesis completeness group was dominated by *Bacteroidota* and *Planctomycetota*, while the high biosynthesis group encompassed a broader diversity of phyla, including *Proteobacteria* and *Actinobacteria* as major contributors. Interestingly, microorganisms with low and high biosynthetic completeness showed no significant difference in relative abundance, possibly due to the small number of taxa in the former group (Fig. 9F).

To better visualize biosynthetic strategies, we projected the data using Uniform Manifold Approximation and Projection (UMAP) (Fig. 9B), which further confirmed the biosynthetic groupings, with strong clustering patterns aligned with biosynthetic capacity. Interestingly, the high biosynthetic group is further separated into two subclusters. One of them, composed of 51 species, was taxonomically diverse yet tightly grouped. We refer to this subcluster as Cluster A, and the remaining genomes from the High biosynthetic group as Cluster B. Cluster A genomes had significantly larger genome sizes than those in Cluster B (Fig. 9H) and appeared to be the main producers of vitamin B12 (Fig. 9G and Supplementary Fig. 14). Cluster B genomes showed higher completeness for vitamin B3.

Finally, among the 57 high-quality genomes from model communities, we observed that we were unable to cultivate any microorganisms from the low biosynthetic group. This might suggest that cultivation techniques to grow them together with their possible host might be needed. While most of the microorganisms we cultivated belonged to the high biosynthetic group (Fig. 9J), the six microorganisms we cultivated from the medium biosynthetic group grew exclusively in groups. Further, a PCA based on only the genomes from the microorganisms we cultivated revealed a different set of clusters with biosynthesis values ranging from 50% to 90% (Fig. 9K, L). Cluster 1, which had the lowest biosynthesis potential, was again mostly composed of species that were capable of growing exclusively in groups. Cultivating a microorganism with lower biosynthetic potential in pure culture might indicate residual amino acids and vitamins in the filtered-sterilized ocean water used as media. Nevertheless, our cultivation strategy still showed that microorganisms with lower anabolic independence prefer to grow in groups.

## Discussion

The field of microbiology has traditionally relied on the isolation of microorganisms from nature in pure culture^44–49^. The practice has yielded foundational insights into microbial physiology^50–52^, metabolism^53–56^, and ecological interactions such as mutualism^28,57,58^ and competition^59–61^. However, this reductionistic approach has limitations, particularly a strong cultivation bias favoring microorganisms with larger genome sizes^41,62^, broader biosynthetic potential^63^, and greater independence. As a result, a substantial portion of the most abundant microbes in natural environments remains uncultivated^64^.

Here, we demonstrate the utility of microbial model communities established through dilution cultivation from a Baltic Sea pelagic sample for obtaining a wide range of previously uncultivated, abundant, and biosynthetically limited microorganisms. Despite using only one type of ocean water as the medium (which did not allow us to control the presence of amino acids or vitamins), our method allowed the cultivation of groups of microorganisms that showcased important ecological principles that govern microbial life in nature. Our results suggest that by increasingly using high-throughput dilution cultivation of microbial model communities, more diverse microorganisms with medium-to-high biosynthesis potential could be cultivated. Moreover, by varying cultivation physicochemical parameters, such as adding catalase^65^, changing light regimes^66^, or temperature^67^, and perhaps by leveraging a better understanding of bacterial host dynamics^68,69^, we will be able to cultivate a greater proportion of the abundant microorganisms found in nature.

Additionally, we found that species growing in microbial model communities composed of more than three species exhibited lower biosynthetic potential for both amino acids and B vitamins compared to species found growing in smaller groups or independently. The reduced per-genome biosynthetic capacity in more complex communities suggests that microorganisms with low anabolic independence are forming metabolic networks to support their nutritional requirements^70^. Interestingly, a previous study has also found a threshold of microbial diversity at which competition and complementation saturate^30^. Specifically, they observed that beyond approximately 26 taxa, further increases in diversity had little detectable impact on overall community function (respiration), suggesting a saturation of functional capacity. Although our work examines only amino acid and vitamin biosynthesis, both studies observed a diversity threshold. These different thresholds observed emphasize that microbial communities likely achieve a balanced state of interaction complexity beyond certain diversity levels, thereby optimizing ecological efficiency.

Our findings also align with the Black Queen Hypothesis, which posits that certain functions, particularly costly biosynthetic pathways, can be lost by some community members as long as the production of the metabolite at the community level is sufficient to sustain the individual^71^. In this context, the reduced genome sizes observed in the abundant species from the model communities and the environment likely reflect a genome streamlining process^72^. The findings further align with the hypothesis that obligate co-existing microbes have evolved to rely on their community for essential nutrients^73^, potentially creating social networks that might bring microbial community stability^14,74–76^. To support this, our stepwise module analysis revealed that microbial model communities with more than three species collectively maintain biosynthetic potential for amino acids and vitamins. These observations also align with recent findings^77^ from a study that examined the potential for metabolic complementarity among auxotrophic soil bacteria. The study analyzed 746 auxotrophic strains from 27 soil-derived communities that were grown in groups of 2, 3, 4, and 5 strains, and described a clear trend: larger groups of bacteria were more capable of collectively producing all necessary amino acids due to metabolic complementarity.

Importantly, our findings also complement large-scale studies investigating the ecological distribution of auxotrophies. A recent analysis of over 26,000 representative bacterial genomes across diverse environments found that amino acid auxotrophy is more common in host-associated environments while relatively rare in aquatic and soil ecosystems. However, this analysis draws a robust but strict boundary on how to categorize microorganisms in a binary model of prototrophs and auxotrophs (more than 40% genes missing per pathway to classify as auxotroph)^16^. While this categorization has been widely used, it might also be hiding potential interdependencies. In our study, we draw no boundary for defining auxotrophy. Instead, we studied the amino acid and B vitamin pathways by observing their completeness and comparing them across the different species in the dataset. We believe this has the potential to reveal a wide spectrum of possibilities where microorganisms might just need a precursor to complete biosynthesis^21^. Moreover, we found further support for the idea that metabolic interdependencies go beyond the simple exchange of end products in a recent study^20^. The authors used 25 engineered strains of *E*. *coli* that were auxotrophic for specific amino acids (arginine, histidine, isoleucine, proline, and tryptophan) and performed pairwise co-cultures of strains auxotrophic for the same amino acid. Strikingly, they found that growth complementation was often achieved by sharing the intermediates within the biosynthetic pathway. For this reason, we believe that moving away from binary categorization of auxotrophies might bring more nuance to the study of anabolic dependencies.

Nevertheless, it is important to acknowledge the limitations inherent to any metagenomic study, including ours. (i) The best-characterized pathways for amino acid and vitamin biosynthesis are based on a few hundred physiologically tested strains. Unknown enzymes and alternative routes likely still exist, even for core amino acids. Taxon-specific variants and incomplete descriptions of alternative steps mean that single gaps in pathways may reflect gaps in knowledge rather than true anabolic dependencies^78^. (ii) Public databases contain significant rates of functional misannotation, mainly because most protein databases are usually annotated automatically using computational approaches^79^. (iii) Despite rigorous quality screening, MAGs remain incomplete; thus, missing steps could represent false negatives. While these limitations apply to our study, the large number of high-quality genomes across diverse taxa that we analyzed, together with the consistency of the observed patterns, gives us confidence that our main conclusions remain robust. Abundant bacteria appear interdependent through essential metabolites, and their cultivation success increases when incubated in groups rather than alone.

In summary, our study highlights the larger potential of microbial model communities in bridging the gap between laboratory cultivation and the environment. By cultivating naturally assembled groups of microorganisms, we recovered ecologically dominant taxa with limited biosynthetic capacity that are often overlooked by traditional isolation techniques. These findings demonstrate that increasing community complexity is associated with different forms of reduced metabolic autonomy. Moreover, we observed that biosynthetic interdependencies can be common among planktonic bacterial taxa and are likely widespread in nature. In combination with recent experimental evidence showing the exchange of biosynthetic intermediates among bacteria, our results reinforce the idea that anabolic dependencies, rather than complete autonomy, are a successful ecological strategy. Cultivating groups rather than individuals can offer a more ecologically relevant understanding of how microbes survive, interact, and evolve in nature.

## Methods

### Sampling and sample processing

We collected an environmental sample from the surface layer of the Baltic Sea close to Askö in the Trosa archipelago (Lat 58°48.20′N, Lon 17°37.42′E) in October 2021 (Fig. 1A). For details on the chemical composition of the water at sampling time, please visit https://shark.smhi.se/hamta-data/. The sample for our experiments was subsequently processed in the laboratory for DNA extraction and metagenomic sequencing. Briefly, the water sample was filtered through a 0.1 µm membrane and used to extract environmental DNA using either the FastDNA® SPIN Kit for Soil (MP Bio) and the Dneasy PowerWater kit (Qiagen). Additionally, we filtered water through a 0.1 µm hollow fiber cartridge (Cytiva) and used the filtrate as media to establish cultures with different starting inoculum sizes.

### Flow cytometry

We used a CytoFLEX instrument manufactured by Beckman Coulter to process the environmental sample and to calculate the cell count as the number of events/mL. Briefly, we stained 50 µL of each sample with Syto13 at a final concentration of 0.025 mM. During the flow cytometer acquisition process, we set the following parameters: FSC 2500, SSC 2500, and FITC 800 with a flow rate of 60 µl/min.

### Establishment of microbial model communities

We used the dilution-to-extinction technique, considering the cell count information from the sample and diluting the cells in the filtered, sterilized water, to achieve approximately the desired starting number of cells in our microbial model communities. The model communities were designed in two ways: low inoculum size and high inoculum size. The low inoculum size model communities included hundreds of individual cultures in 96-well plates with a starting number of cells of 0 (control), 2, 6, 10, 15, 20, 30, 50, and 100 cells/well. The high inoculum size model communities were prepared in bottles with starting numbers of cells of 0 (control), 200, 600, 1000, 1500, 2000, 3000, 5000, 10,000, 100,000, and 1,000,000 cells/bottle. Filtered water from the original environmental sample, without any additional nutrients, was used as the medium, resulting in an undefined medium closely reflecting in situ conditions. All cultures were incubated with light/dark cycles (light 6:42 h, dark 18:32 h each day) and 12.2 °C at light and 11.8 °C at dark for 4 weeks before further processing. This regime was designed to emulate as best as possible the natural fluctuations in time, temperature, and light that cells experience in their environment.

### MDA, library preparation, and sequencing

Before sending our samples for metagenomic sequencing, we performed MDA on all cultures to increase the concentration of DNA. The MDA reaction consisted of 0.6 µL of culture and 4.4 µL of reaction mix using the Repli-g Single Cell kit (Qiagen). After DNA amplification, we found that 315 of the cultures passed the amplification threshold of the negative controls set in the MDA reaction. These cultures were deemed positive and were sent for sequencing. We extracted DNA from the selected cultures and our environmental sample with two different DNA extraction methods and subjected them to library preparation using the TruSeq PCR-Free DNA library preparation kit (Illumina Inc.), followed by metagenomic Illumina sequencing at the SNP&SEQ Platform at Uppsala University. This sequencing technology utilized cluster generation and 150 cycles of paired-end sequencing on an SP flow cell, employing the NovaSeq 6000 system with v1.5 sequencing chemistry (Illumina Inc).

### Genome-resolved metagenomics pipeline

We removed low-quality reads from the raw sequences using the software Trimmomatic (v0.36)^80^ with the following options: ILLUMINACLIP:TruSeq3-PE-2.fa:2:30:10:2:keepBothReads LEADING:3 TRAILING:3 SLIDINGWINDOW:4:15 MINLEN:50. We employed the MetaWRAP pipeline (v1.3.2)^81^ to process our cleaned metagenomic reads. First, clean reads were assembled in a single-sample assembly style using the “metaWRAP_assembly” module with MegaHit (v1.1.3)^82^ for the environmental samples and with SPAdes (v3.15.3)^83^ for the culture samples. The quality of the assemblies was assessed with QUAST (v.5.0.2)^84^. Since the culture DNA was amplified using MDA, read-coverage information could not be used. For this reason, we included background metagenomic data from previous projects in the Baltic Sea (Fig. 1A and Supplementary Data 2) for binning. Subsequently, we used multiple-sample coverage binning to decrease the contamination and increase the completeness of bins^85^. The reads were mapped against all assemblies using the Input_POGENOM pipeline^86^, which uses Bowtie2^87^ with default parameters. After mapping and obtaining the BAM files, the minimum coverage was calculated using samtools (v1.9)^88^. Only coverage values for each assembly in each metagenomic sample with mean coverage ≥20× and mean breadth ≥40% were retained, following Input_POGENOM recommendations. These coverage values for each sample were combined and processed with the “metaWRAP_binning” module, which uses three metagenomic binning tools: metaBAT2^89^, maxBIN2^90^, and CONCOCT^91^. We consolidated all the bins generated by these different tools using the “metaWRAP_bin_refinement” module. We classified the resulting bins taxonomically with GTDB-Tk (v2.1.1)^92^. Finally, we assessed the quality of the bins using CheckM (v1.1.3)^93^. We considered bins as MAGs when they had a completeness of >45% and a contamination of <10%, and these MAGs were included for further analysis.

### Complementing with previously published MAGs

The dereplicated MAGs obtained here were supplemented with 771 MAGs from an earlier study^37^ that were based on metagenomics data from three studies^37–39^. We dereplicated the collection of MAGs to obtain species-cluster representatives using ANI > 95% with mOTUpan (v0.3.2)^94^, and selected the genome with the highest quality as the species-cluster representative genome.

### Relative abundance analysis

To calculate the relative abundance of our 701 species-clusters in the BalticMAG catalog, we employed the mapping tool Strobealign (v0.14.0)^95^, which aligned the short metagenomic reads to our species-cluster collection using a high-speed indexing method (Supplementary Data 5). Briefly, we created three different indexes with different lengths (100, 125, and 150) for our 701 species clusters. We filtered out low-quality reads from our 112 environmental samples with Trimmomatic (v0.36)^80^ with the following options: ILLUMINACLIP:TruSeq3-PE-2.fa:2:30:10:2:keepBothReads LEADING:3 TRAILING:3 SLIDINGWINDOW:4:15 MINLEN:50. After this, we performed a competitive mapping of all reads against our created index to obtain the corresponding BAM file for each sample. We sorted the BAM files with the Anvi’o platform (v7.1)^96^ using the “anvi-init-bam” program, and we calculated the coverage of each genome per sample with the program “anvi-profile-blitz.” We calculated each species-cluster’s relative abundance with the previously obtained output by dividing each genome’s mean coverage inner quartiles (i.e., q2q3_cov) by the overall sample mean coverage. We also computed the prevalence, defined as the frequency of each species-cluster across samples. Specifically, prevalence represents the proportion of samples in which a species-cluster was detected with a relative abundance > 0.

### Custom functional annotation of KEGG Orthologs (KOs) and biosynthetic modules

We used the Anvi’o platform (v7.1)^97^ to perform functional annotation of KEGG Orthologs (KOs) and to estimate metabolic potential. Initially, for each genome, we used “anvi-gen-contigs-database” to create a contigs database, which served as the basis for the subsequent functional annotation steps. To annotate each genome with KOs from the KEGG KOfam database^98^, we ran the “anvi-run-kegg-kofams” program. We then predicted the metabolic capabilities of each genome by running the “anvi-estimate-metabolism” program^99^ (Supplementary Data 6).

In addition to the default KEGG modules^100,101^, we developed and implemented a custom set of 30 curated modules targeting the biosynthesis of amino acids and B vitamins (Supplementary Data 7–9). Our pathway curation is focused on the KEGG database to ensure a single, consistent framework across amino acids and B vitamins, thereby ensuring scalability and reproducibility within Anvi’o across hundreds of genomes. KOs were selected directly from the KEGG pathway map for each metabolite, and we encoded explicit logical rules (OR/AND) to represent alternative branches within a single, unified route. Where KEGG provides multiple modules that yield the same end-product, we have consolidated them into a single custom module (e.g., cysteine from serine via KEGG module M00021 or M00338). For metabolites lacking a KEGG module, we defined a completely new custom route by selecting the relevant KOs directly from the KEGG pathway map for that metabolite and applying the same OR/AND logic. Custom module IDs use the suffix _00 for consolidated versions of multiple pathways for a given metabolite, whereas _01 distinguishes alternative variants when relevant (e.g., aerobic vs. anaerobic vitamin B12 biosynthesis). Custom module definitions and implementation files are publicly available in the accompanying GitHub repository: https://github.com/ivagljiva/custom_biosynthesis_modules.

Custom modules were integrated using the “anvi-setup-user-modules” command. Completeness scores for each genome and custom module were calculated using “anvi-estimate-metabolism” with the “--only-user-modules” flag. Although we initially created custom modules for all 20 proteinogenic amino acids (plus the important precursor chorismate), we subsequently excluded alanine, asparagine, and aspartate modules from downstream analysis. These three amino acids are commonly produced by generic transamination reactions with central metabolic intermediates (e.g., pyruvate or oxaloacetate), and their biosynthesis often involves multiple redundant enzymes that are still challenging to annotate accurately^102^. Because our custom definitions included only a very limited subset of these enzymes, we observed artificially low completeness scores for these three modules. Thus, for accuracy and consistency, we retained only 18 amino acid modules (plus the 9 B vitamin modules) for downstream statistical comparisons (Supplementary Data 10).

### Statistics and reproducibility

All statistical analyses from this study were performed with R software (v4.4.0)^103^ and RStudio^104^. We used the Shapiro test to assess the normality of our data to be compared; if *p* < 0.05, we interpret this as not normally distributed^105^. Since our data were not normally distributed, we employed a non-parametric test, such as the Wilcoxon test, to find differences between pairs of groups (e.g., culture vs. environment)^106^. To assess statistical differences among more than two groups, we first applied a Kruskal–Wallis rank-sum test to determine if any group differed significantly^107^. When the Kruskal–Wallis test was significant (*p* < 0.05), we performed post hoc pairwise comparisons using Dunn’s test with Bonferroni correction for multiple testing^108^. To visualize pairwise group differences, we applied a compact letter display, where groups that do not differ significantly share the same letter, and groups with different letters are significantly different from each other. Associations were evaluated using Spearman’s rank correlation method, a non-parametric statistic suitable for our data, which is not normally distributed. The correlation coefficient (*ρ*) and *p* value are provided. A trend line is included in the correlation plots for visualization purposes only.

Finally, variation in biosynthetic potential among high-quality genomes was explored using PCA of the biosynthetic completeness matrix. To visualize overall similarity patterns, we also applied UMAP to the same matrix. Clustering patterns were further assessed with k-means clustering (*k* = 3) based on the first five PCA components.

### Reporting summary

Further information on research design is available in the Nature Portfolio Reporting Summary linked to this article.

## Supplementary information

Supplementary Information

Description of Additional Supplementary Materials

Supplementary Data 1–12

Reporting Summary

## Data Availability

The paired-end sequences of both environmental (*n* = 2) and culture (*n* = 204) metagenomic samples from the Baltic Sea collected in this study, along with the corresponding 827 MAGs (>45% completeness and <10% contamination), are publicly available in the NCBI under the BioProject ID PRJNA1134408. The data can be accessed at https://www.ncbi.nlm.nih.gov/bioproject/PRJNA1134408. All other metagenomes from the Baltic Sea (*n* = 110) were downloaded from public repositories, and their metadata are included in Supplementary Data 2, along with their publication references. Source data and categories for salinity concentration (expressed as PSU; equivalent to ‰) used to plot Fig. 1B are provided in Supplementary Data 2. Data for Fig. 1E are derived from Supplementary Data 5 (relative abundance) in combination with metadata in Supplementary Data 4 (culture, environment, or both). Data for Fig. 2 are available in Supplementary Data 4 (metadata and genome source information). Data for Fig. 4 are in Supplementary Data 4 (culture ID and community complexity categories). Data for Fig. 5B, E, F are in Supplementary Data 4 (group, single, or both; “singleton-capable” combines the single and both categories). Data for Fig. 6B, D are in Supplementary Data 11 (pathway completeness for the high-quality cultivated genomes, *n* = 305, including their community complexity category, 1, 2, 3, or more than 3 species per culture). Data for Fig. 9C–I, L are in Supplementary Data 12 (cluster assignments 1–3 correspond to groups shown in Fig. 9K, L).

## Code availability

Custom module definitions and implementation files are publicly accessible at https://github.com/ivagljiva/custom_biosynthesis_modules and archived on Zenodo under 10.5281/zenodo.17465601^109^.
