# Supplementary material for: Microbial model communities exhibit widespread metabolic interdependencies
Source: Commun Biol. 2025 Dec 3;8:1752. doi: 10.1038/s42003-025-09306-y (PMC12680743; doi:10.1038/s42003-025-09306-y)
Supplement: Supplementary file 1 — Supplementary Information [file 42003_2025_9306_MOESM1_ESM.pdf]

SUPPLEMENTARY FIGURES: Microbial model communities exhibit widespread metabolic interdependencies.

Armando Pacheco-Valenciana[1*], Anna Tausch[2], Iva Veseli[3,4], Jennah E. Dharamshi[1], Fabian Bergland[1], Luis Fernando Delgado-Zambrano[5], Alejandro Rodríguez-Gijón[1], Anders F. Andersson[5], Sarahi L. Garcia[1,2,3*].

[1] Department of Ecology, Environment, and Plant Sciences, Science for Life Laboratory, Stockholm University, Stockholm, Sweden.
[2] Institute for Chemistry and Biology of the Marine Environment (ICBM), School of Mathematics and Science, Carl von Ossietzky Universität Oldenburg, 26129, Oldenburg, Germany
[3] Helmholtz Institute for Functional Marine Biodiversity at the University of Oldenburg (HIFMB), Oldenburg, Germany
[4] Alfred Wegener Institute, Helmholtz Centre for Polar and Marine Research, 27570 Bremerhaven, Germany
[5] Department of Gene Technology, Science for Life Laboratory, KTH Royal Institute of Technology, Stockholm, Sweden.

* Correspondence: armando.pacheco@su.se, sarahi.garcia@su.se

KEYWORDS: microbial model communities, metagenomics, anabolic dependencies, microbial interdependencies.

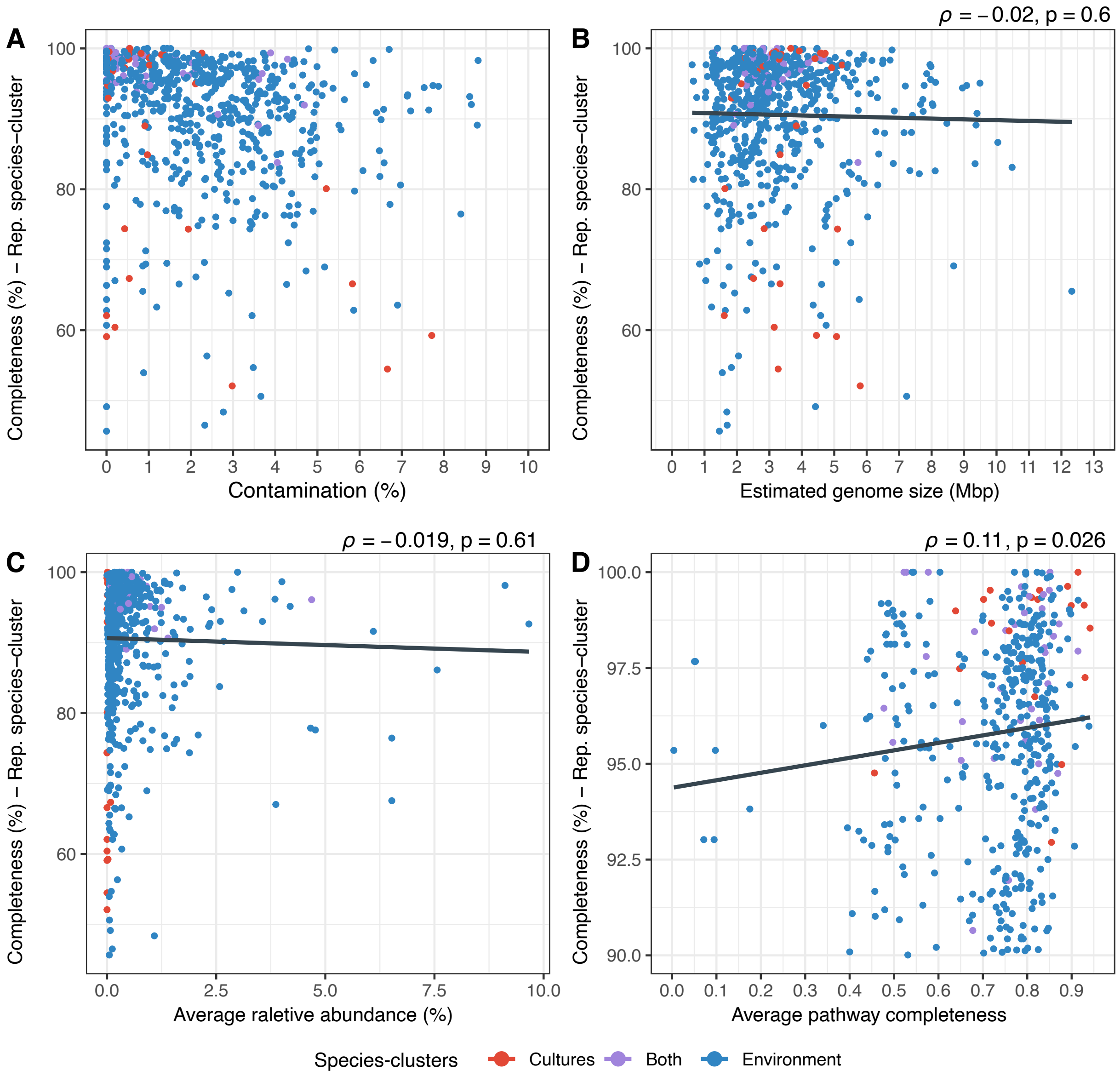

Supplementary Figure 1. Quality metrics of BalticMAG species-cluster representatives. Dot plots showing the relationship between genome completeness and various genomic metrics across the BalticMAG catalog (n = 701): (A) contamination, (B) estimated genome size, (C) average relative abundance, and (D) average pathway completeness for amino acids and vitamins. For panel D, only genomes with >90 % completeness and <5 % contamination were included (n = 450). Each data point represents one species-cluster representative genome found exclusively in model communities (red), exclusively in the environment (blue), or in both (purple). Spearman's rank correlations are shown (ρ, p); trend lines are for visualization only. Publicly available Baltic Sea metagenomes were included in the analysis[37–39].

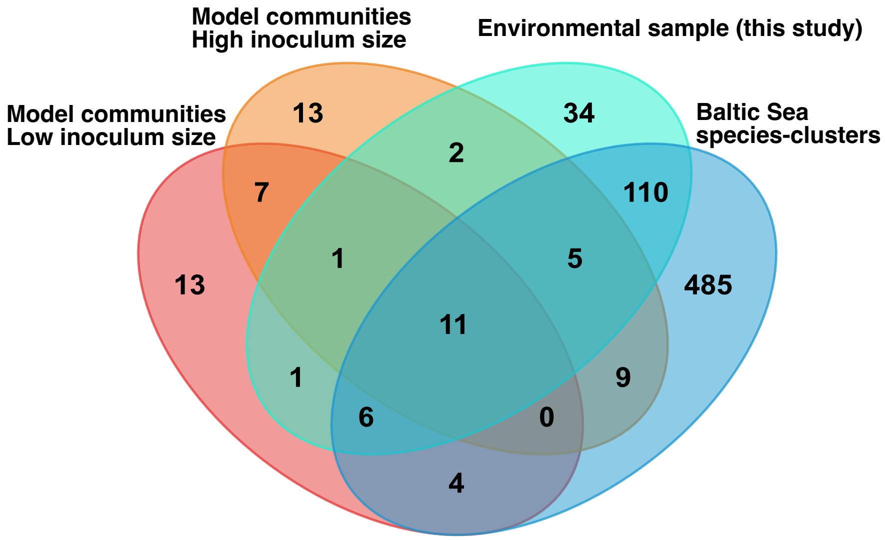

Supplementary Figure 2. Overlap of the BalticMAG species-clusters across different sources. Venn diagram illustrating the overlap of species-clusters from model communities (low inoculum = red, high inoculum = orange), environmental metagenomes from this study (light blue), and publicly available Baltic Sea metagenomes (dark blue)[37–39]. The diagram shows the extent of overlap among these groups.

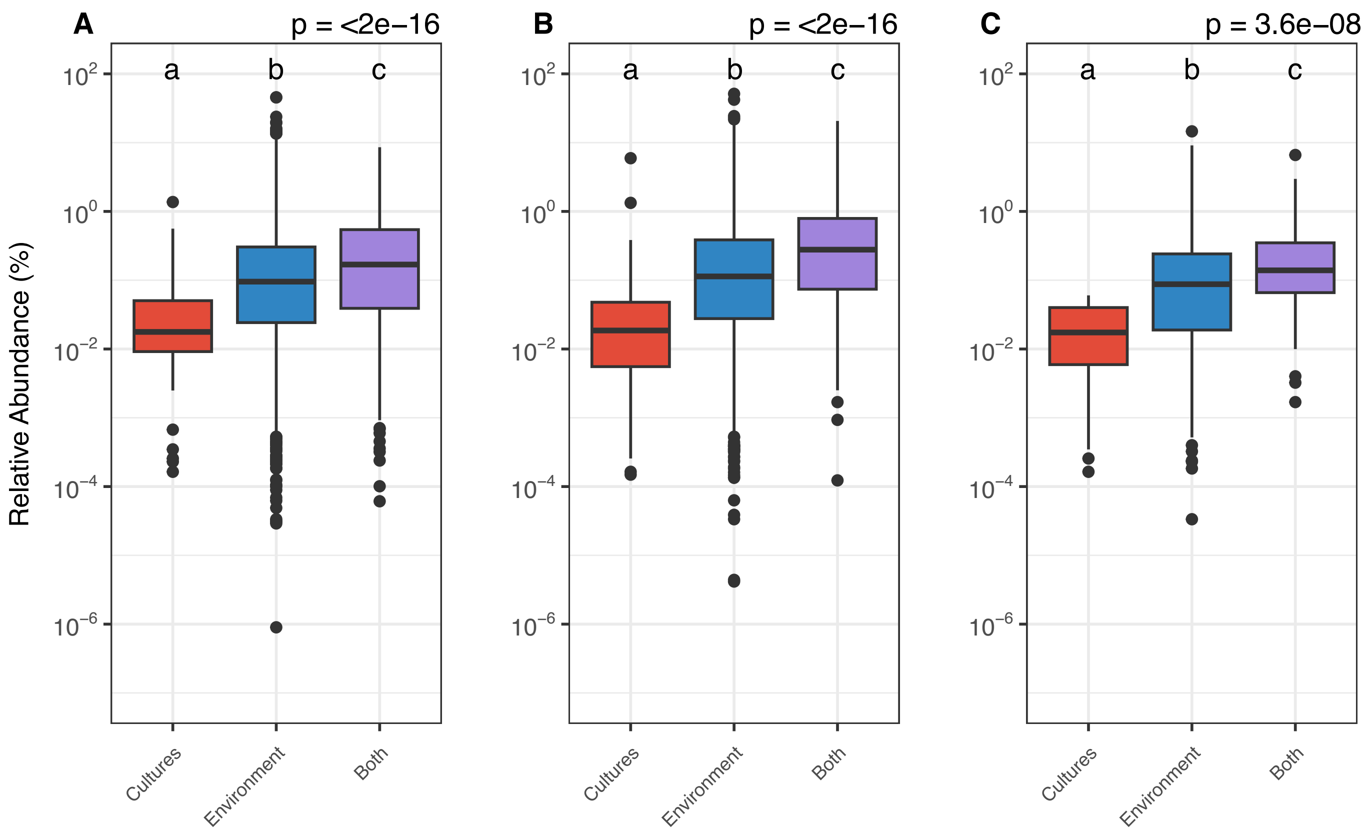

Supplementary Figure 3. Relative abundance of species-clusters in cultures and the environment. Boxplots comparing the relative abundance of species-clusters (n = 701) by their presence in cultures (red), the environment (blue), or both (purple). (A) Relative abundances of all species across all samples with salinity between 7 and 8 ‰ (PSU) and (B) across only the samples from the same location as those used to establish model communities. (C) Relative abundances of species only detected in our own metagenomic samples (n = 566), compared across all metagenomic samples. Statistical significance was tested using the Kruskal–Wallis test followed by Dunn's post hoc test with Bonferroni correction. Groups sharing at least one letter (e.g., a and ab) are not significantly different; groups with different letters (e.g., a vs b) differ significantly ($p < 0.05$). Boxplots show the median and interquartile range; whiskers represent 1.5 × IQR. Publicly available metagenomes were included in the analysis[37–39].

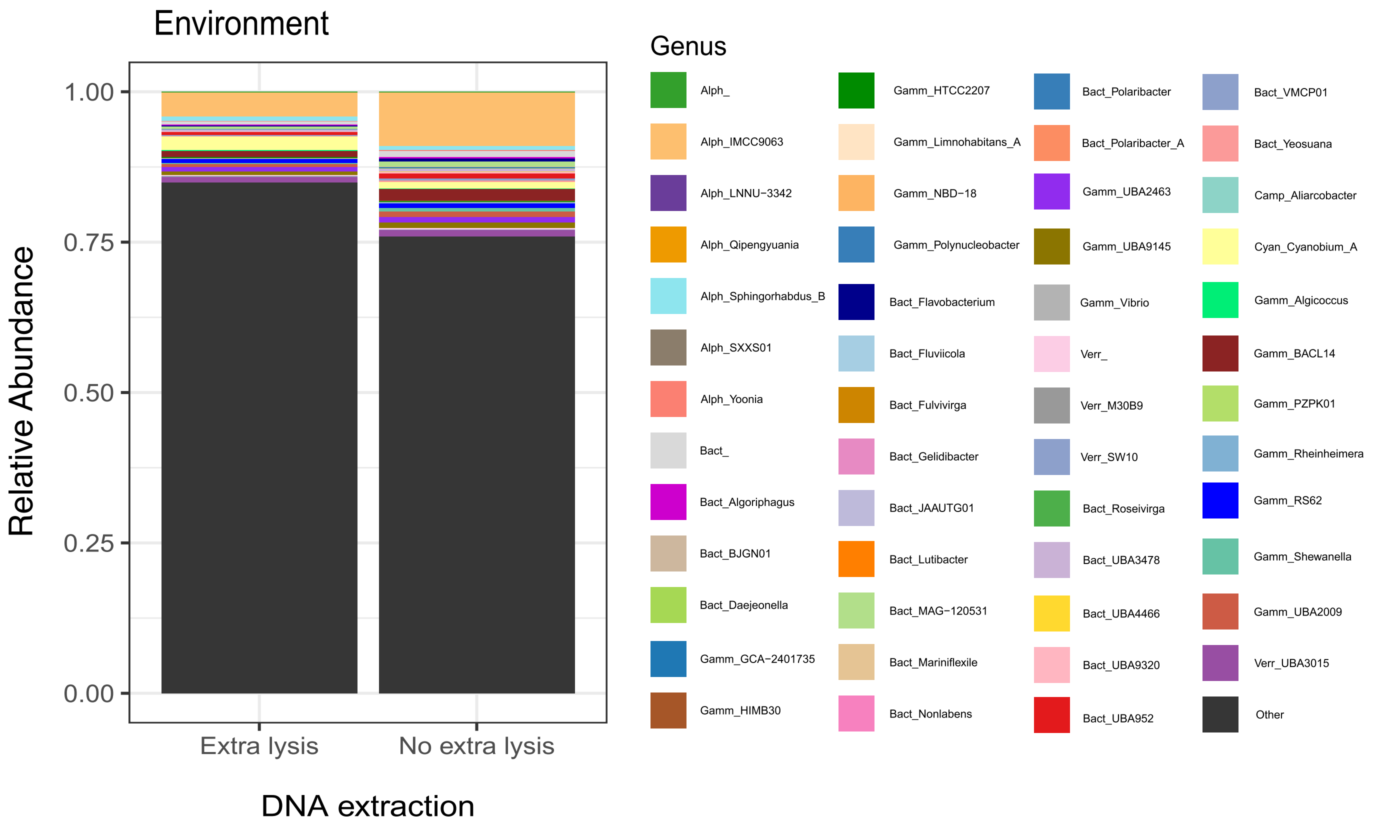

Supplementary Figure 4. Relative abundance of cultured and environmental genomes in the sample of origin. Barplot showing the relative abundance of environmental (dark grey) and cultivated (other colors) genomes in the sample of origin of the cultures (n = 2). Note that the sample was extracted with two different DNA extraction methods, leading to two different perspectives on the diversity. Genomes are color-coded by genera, and the legend shows abbreviated prefixes indicating the class of each genus (e.g., Alph_ for Alphaproteobacteria, Bact_ for Bacteroidia, Camp_ for Campylobacteria, Cyan_ for Cyanobacteria, Gamm_ for Gammaproteobacteria, and Verr_ for Verrucomicrobiae.

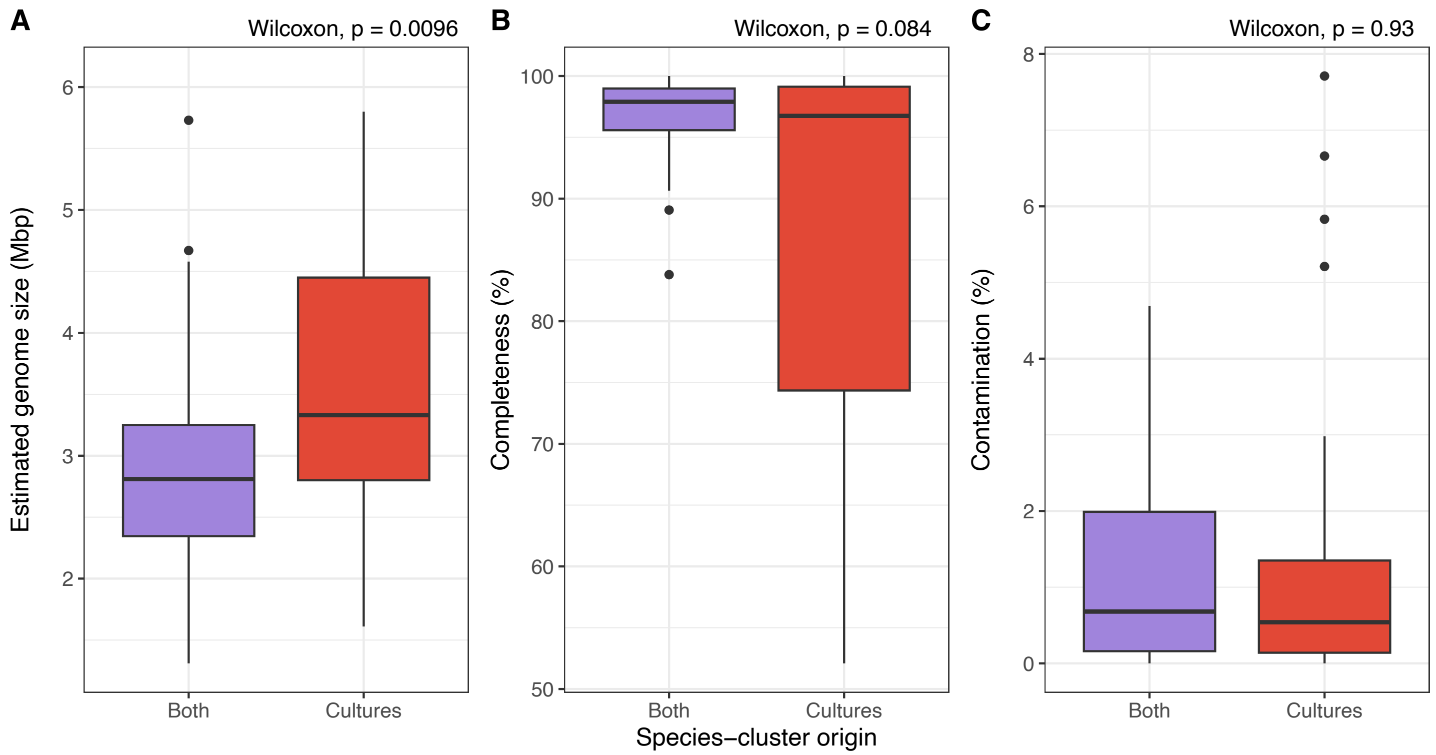

Supplementary Figure 5. Genome metrics of species-clusters from different sources. Boxplot comparing the (A) estimated genome size (Mbp), (B) completeness, and (C) contamination of species-clusters found only in cultures (red, n = 33), and in both cultures and the environment (purple, n = 39). Statistical significance was tested using the Wilcoxon rank-sum test ($p < 0.05$). Boxplots show the median and interquartile range; whiskers represent 1.5 × IQR.

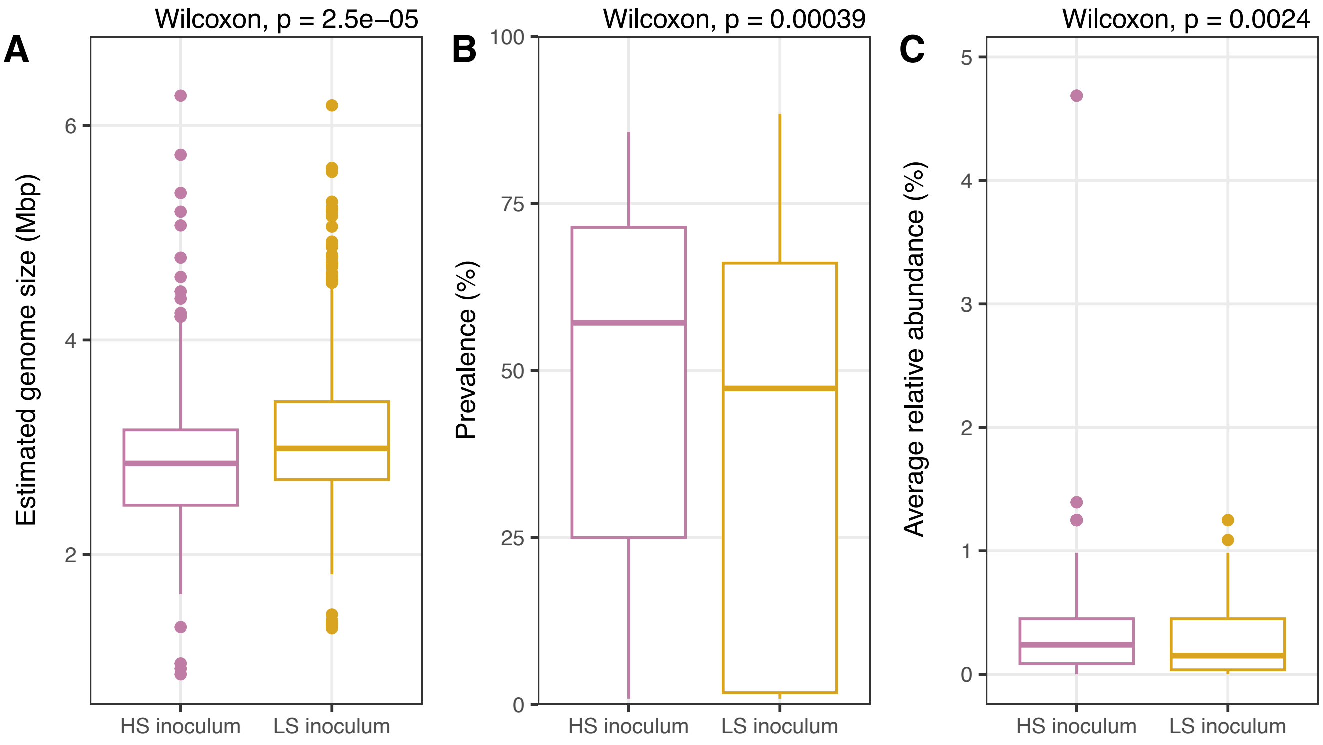

Supplementary Figure 6. Genomic features of MAGs from model communities from high and low inoculum sizes. Boxplots comparing the (A) estimated genome size, (B) prevalence, and (C) average relative abundance of genomes from model communities with a high inoculum size (HS, n = 231) and a low inoculum size (LS, n = 296). Statistical significance was tested using the Wilcoxon rank-sum test ($p < 0.05$). Boxplots show the median and interquartile range; whiskers represent 1.5 × IQR.

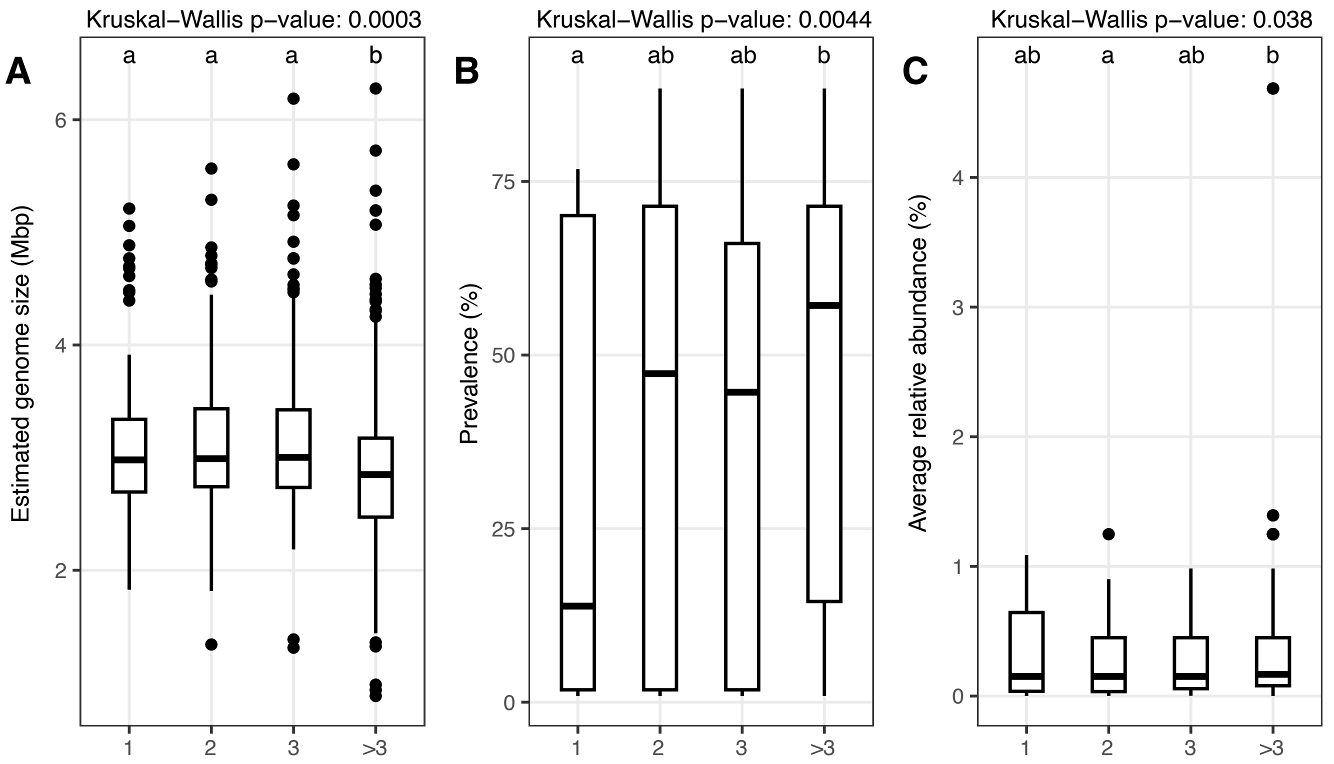

Supplementary Figure 7. Genomic features of cultured genomes across community complexity. Boxplots comparing the (A) estimated genome size, (B) prevalence, and (C) average relative abundance of genomes from model communities (n = 527) that include 1, 2, 3, or more than 3 species per culture. Statistical significance was tested using the Kruskal–Wallis test ($p < 0.05$), followed by a Dunn's post hoc test with Bonferroni correction. Groups sharing at least one letter (e.g., a and ab) are not significantly different; groups with different letters (e.g., a vs b) differ significantly ($p < 0.05$). Boxplots show the median and interquartile range; whiskers represent 1.5 × IQR.

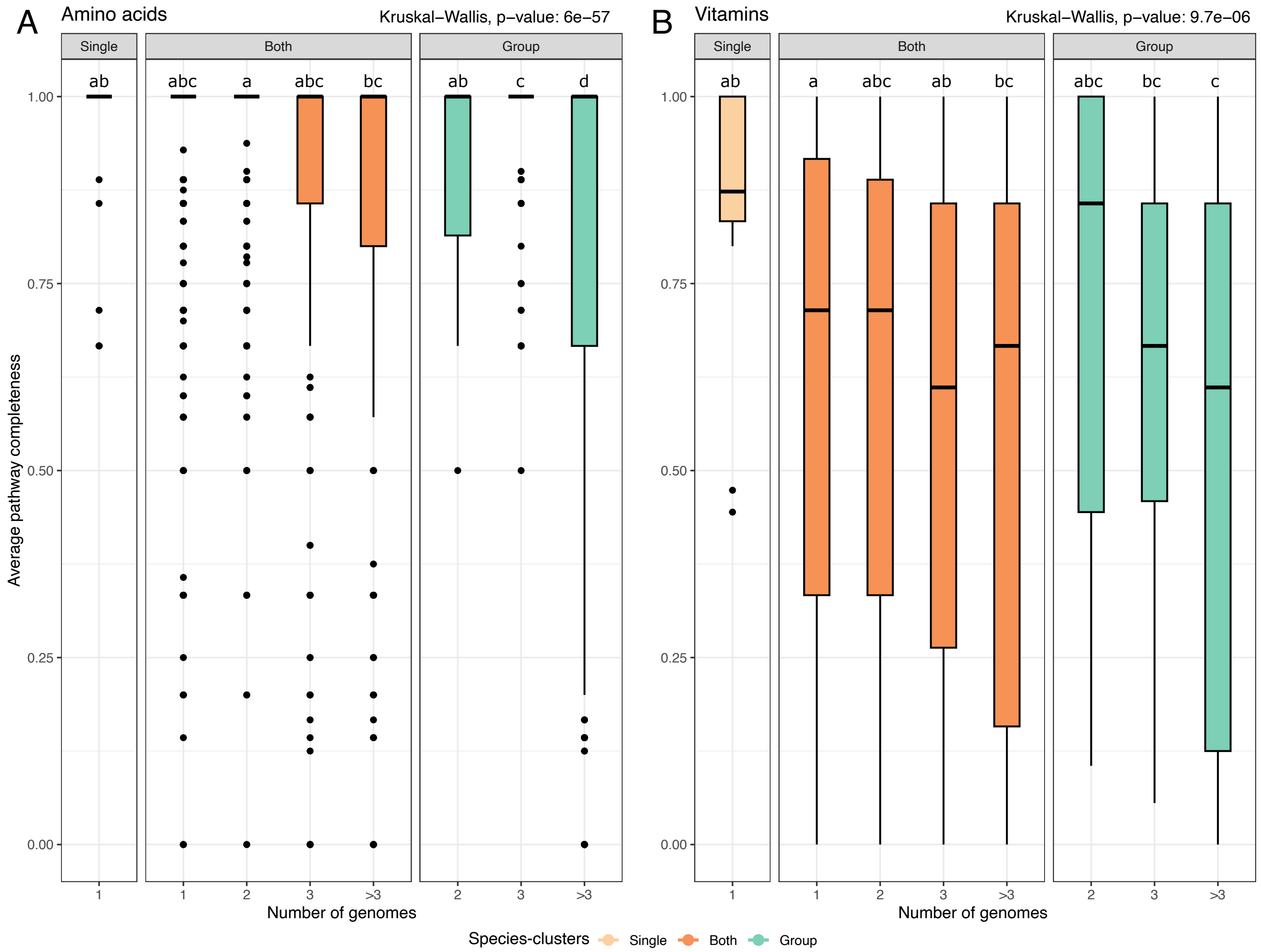

Supplementary Figure 8. Pathway completeness of cultured genomes according to growth categories and community complexity. Boxplots showing the average pathway completeness of cultured high-quality genomes (n = 305, >90 % completeness and <5 % contamination) for (A) custom amino acid and (B) vitamin modules, according to the number of genomes per culture, as well as the growth categories. Color coding indicates whether the species cluster grew exclusively on its own (light orange), exclusively in groups (green), or both on its own and in groups (orange). Statistical significance was tested using the Kruskal–Wallis test ($p < 0.05$), followed by a Dunn's post hoc test with Bonferroni correction. Groups sharing at least one letter (e.g., a and ab) are not significantly different; groups with different letters (e.g., a vs b) differ significantly ($p < 0.05$). Boxplots show the median and interquartile range; whiskers represent 1.5 × IQR.

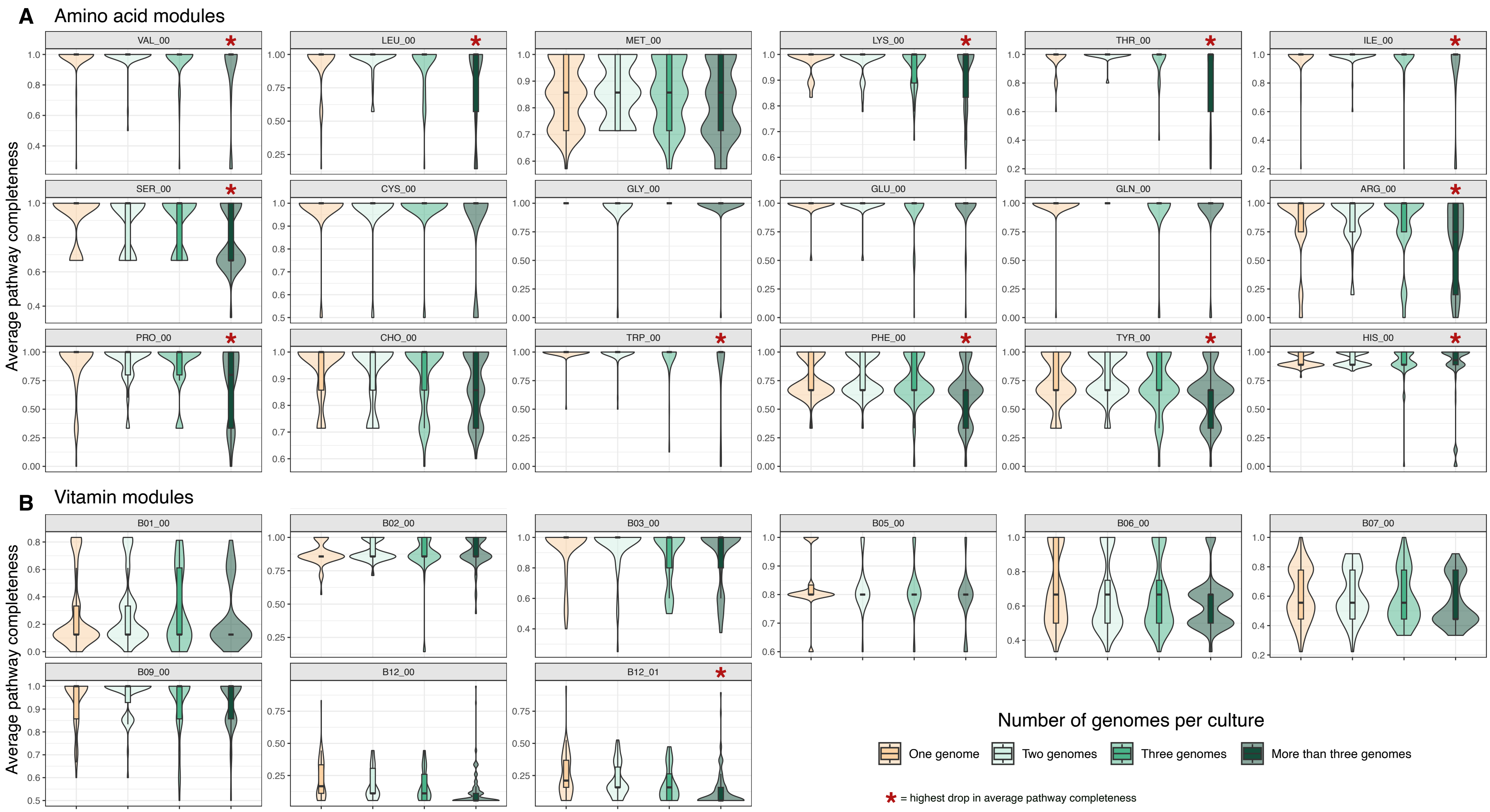

Supplementary Figure 9. Module completeness patterns of cultured genomes across community complexity. (A) Violin plots showing average pathway completeness for custom amino acid and (B) vitamin modules. Each point represents one genome (n = 305, >90 % complete, <5 % contaminated) from model communities, compared by community complexity: 1 (light orange), 2 (mint), 3 (green), and >3-species cultures (dark green). Red asterisks indicate modules with the largest relative decreases in completeness in >3-species communities compared to simpler communities.

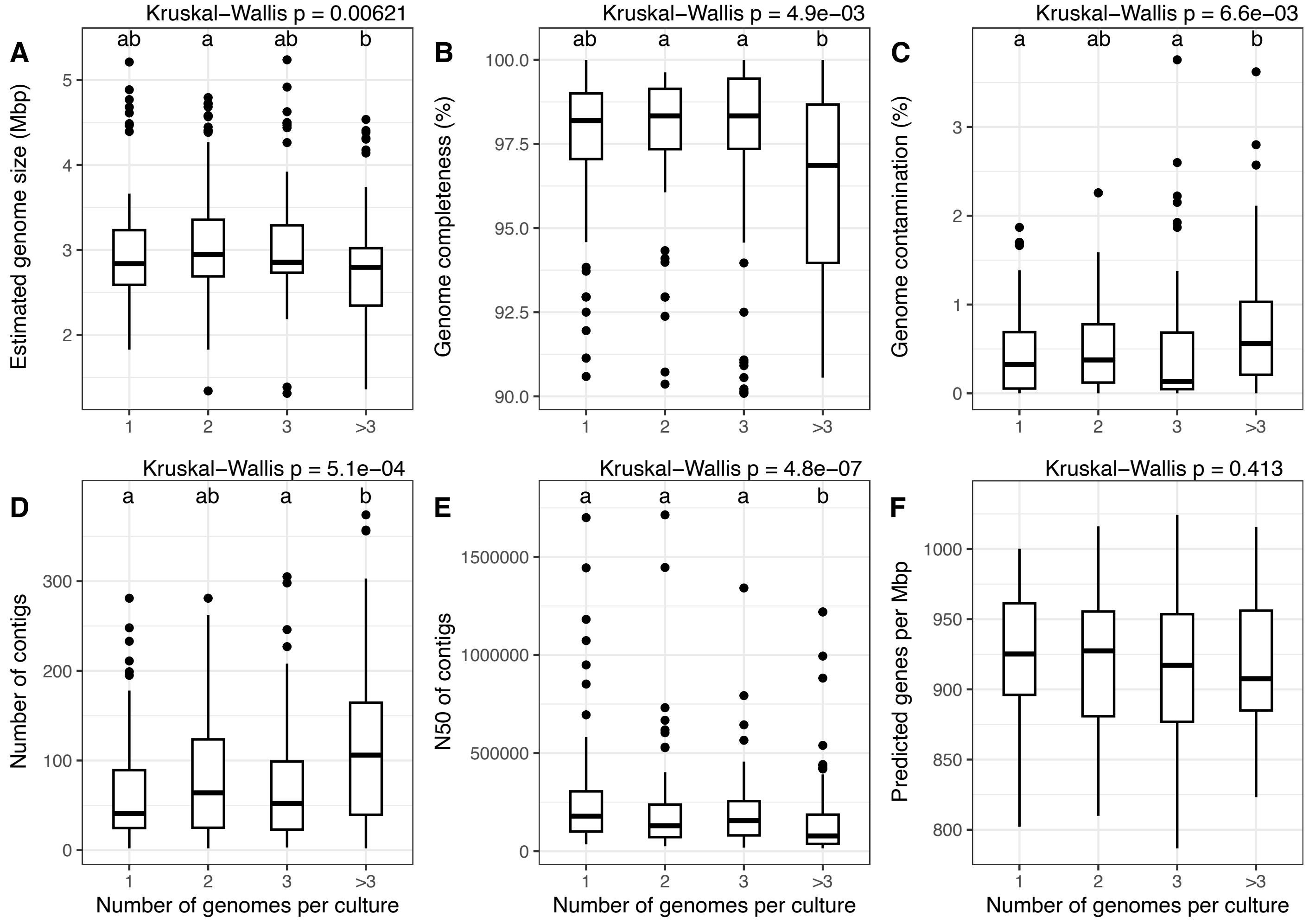

Supplementary Figure 10. Genomic quality metrics of cultured genomes across community complexity. Boxplots comparing high-quality genomes (n = 305, >90 % complete, <5 % contaminated) for (A) estimated genome size, (B) genome completeness, (C) genome contamination, (D) number of contigs per genome, (E) N50 of contigs, and (F) predicted genes per Mbp across communities with 1, 2, 3, or >3 species per culture. Statistical significance was tested using the Kruskal–Wallis test ($p < 0.05$), followed by a Dunn's post hoc test with Bonferroni correction. Groups sharing at least one letter (e.g., a and ab) are not significantly different; groups with different letters (e.g., a vs b) differ significantly ($p < 0.05$). Boxplots show the median and interquartile range; whiskers represent 1.5 × IQR.

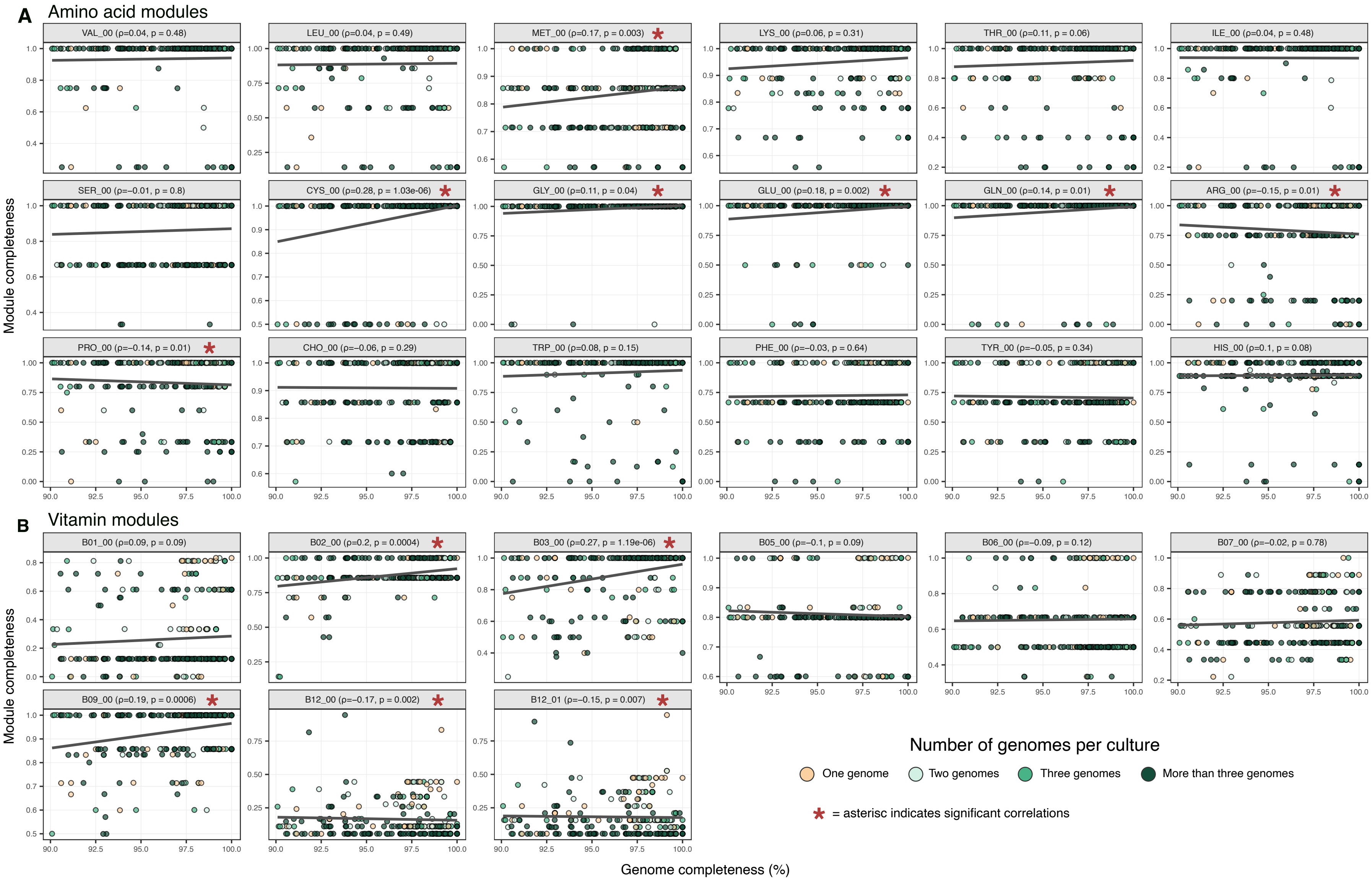

Supplementary Figure 11. Relationship between genome completeness and module pathway completeness in cultured genomes. Scatter plots for (A) custom amino acid biosynthesis modules, and (B) vitamin biosynthesis modules. Each point represents one genome (n = 305, >90 % complete, <5 % contaminated), color-coded by community complexity: 1 (light orange), 2 (mint), 3 (green), and >3 species per culture (dark green). Spearman's rank correlations are shown (ρ, p), and trend lines are included for visualization only. Red asterisks indicate modules with significant correlations.

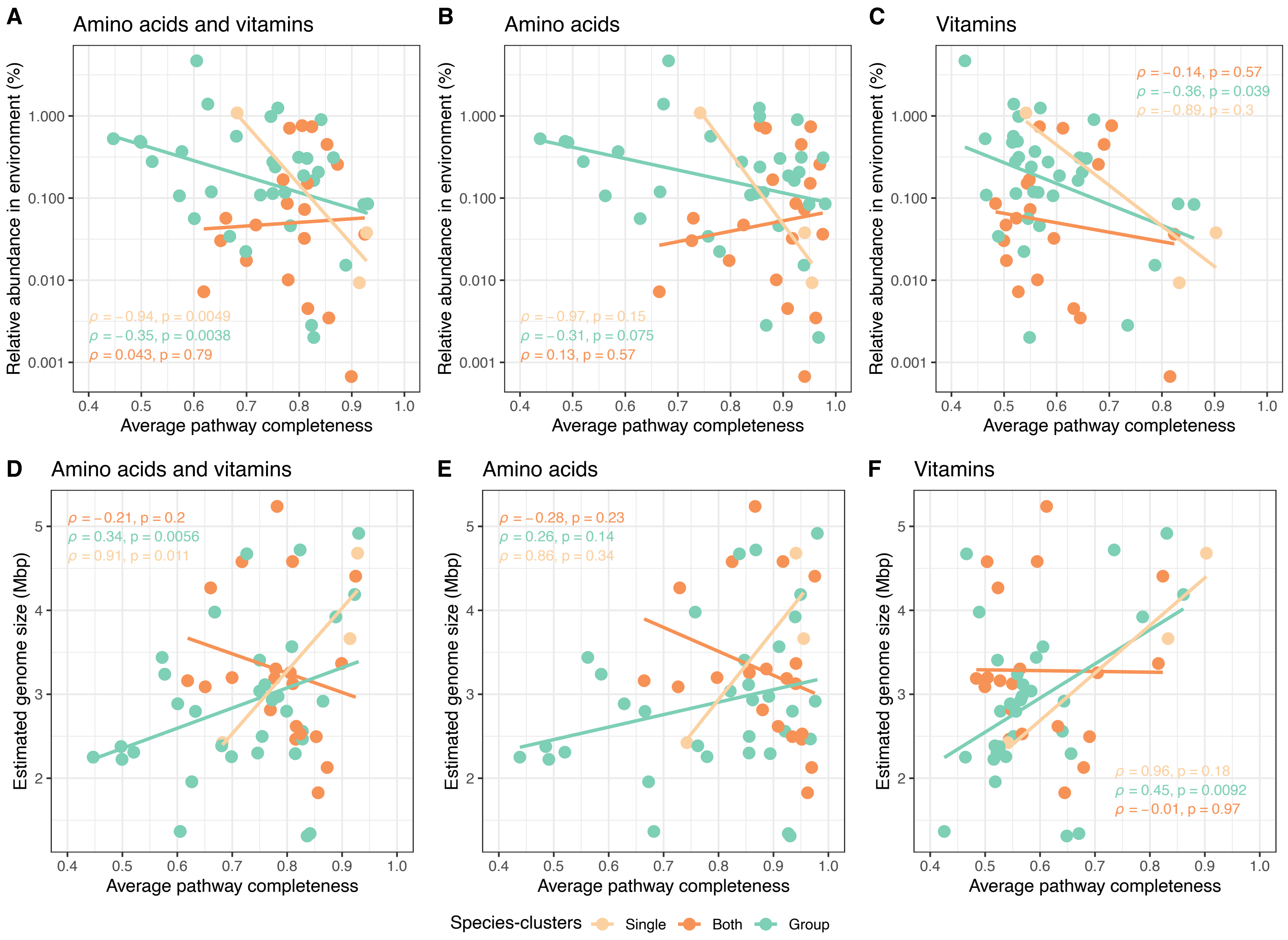

Supplementary Figure 12. Relationship between pathway completeness, relative abundance, and genome size of cultured species-clusters. Scatter plot showing the relationship between average biosynthesis pathway completeness and relative abundance (A, B, and C) and estimated genome size (D, E, and F) for three metabolic categories: Amino acids and vitamins (A, D), only amino acids (B, E), and only vitamins (C, F). Each data point represents a species-cluster (n = 57, >90 % completeness, <5 % contamination), which either grew exclusively on its own (light orange), exclusively in groups (green), or both on its own and in groups (orange). Spearman's rank correlations are shown (ρ, p).

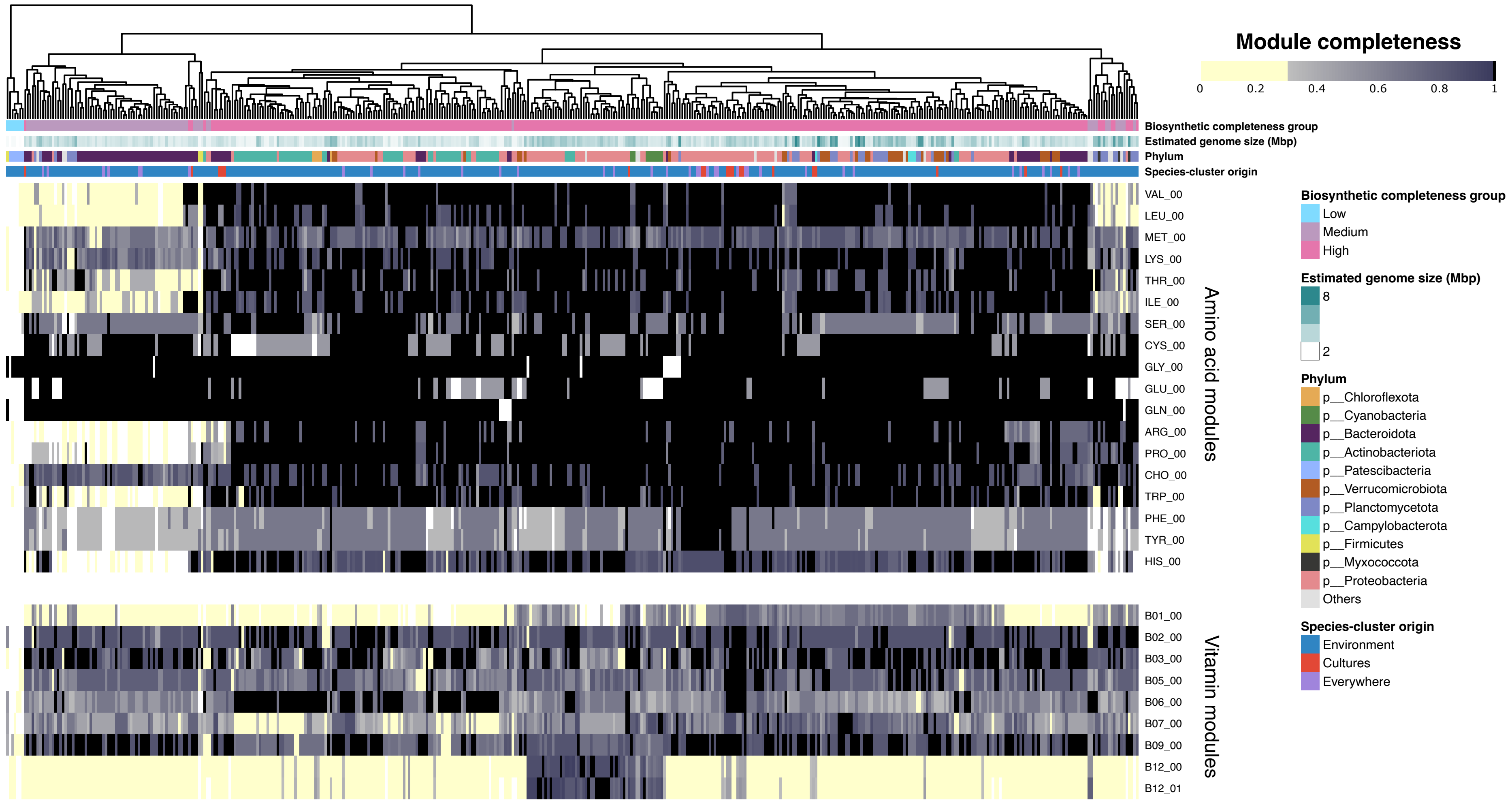

Supplementary Figure 13. Biosynthetic potential clustering of the BalticMAG catalog. Heatmap showing the pathway completeness of amino acid and vitamin biosynthesis pathways (based on KEGG custom modules) across high-quality species-clusters (n = 450, >90 % completeness, <5 % contamination). The color gradient represents pathway completeness, with white = 0 %, yellow = >0–30 %, and black = 100 % completeness. Genome annotations include biosynthetic completeness group (Low = light blue, Medium = light purple, High = pink), estimated genome size (white to dark teal gradient), taxonomic affiliation (color-coded by phylum), and genomic origin (environment-only = blue, culture-only = red, both = purple). Columns are clustered based on similarity in biosynthetic completeness profiles. Publicly available metagenomes were included in the analysis[37–39].

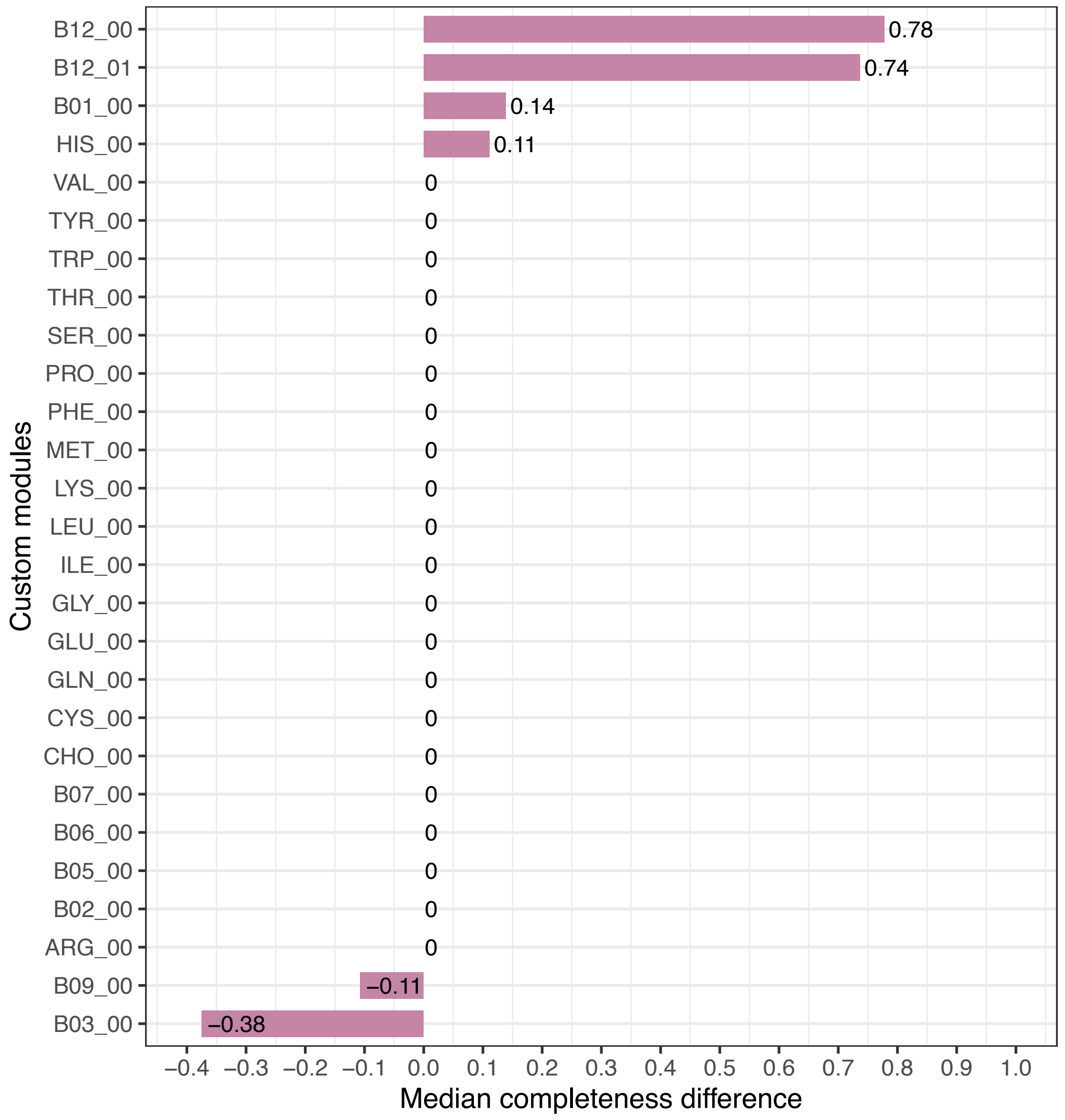

Supplementary Figure 14. Differences in biosynthetic module completeness between Cluster A and B genomes. Median completeness differences for KEGG custom biosynthesis modules between Cluster A (n = 51) and Cluster B (n = 308) genomes from the high biosynthetic completeness group. Positive values indicate that modules are more complete in Cluster A, negative values indicate that they are more complete in Cluster B, and zero values indicate no difference in median completeness between the two clusters.

SUPPLEMENTARY REFERENCES for Supplementary Data 1

1. Peng, H. *et al.* A molecular toolkit of cross-feeding strains for engineering synthetic yeast communities. *Nat Microbiol* **9**, 848–863 (2024).
2. Carrasco Flores, D. *et al.* A mutualistic bacterium rescues a green alga from an antagonist. *Proc. Natl. Acad. Sci. U.S.A.* **121**, e2401632121 (2024).
3. Pinheiro, Y. *et al.* A thermophilic chemolithoautotrophic bacterial consortium suggests a mutual relationship between bacteria in extreme oligotrophic environments. *Commun Biol* **6**, 230 (2023).
4. Liu, Y. A three-species microbial consortium for power generation. *Environmental Science* (2017).
5. Brooks, S. M. *et al.* A tripartite microbial co-culture system for de novo biosynthesis of diverse plant phenylpropanoids. *Nat Commun* **14**, 4448 (2023).
6. Beiralas, R., Ozer, N. & Segev, E. Abundant *Sulfitobacter* marine bacteria protect *Emiliania huxleyi* algae from pathogenic bacteria. *ISME Communications* **3**, 100 (2023).
7. Jung, D. *et al.* Accessing previously uncultured marine microbial resources by a combination of alternative cultivation methods. *Microbial Biotechnology* **14**, 1148–1158 (2021).
8. Zhang, X. & Reed, J. L. Adaptive Evolution of Synthetic Cooperating Communities Improves Growth Performance. *PLoS ONE* **9**, e108297 (2014).
9. Harcombe, W. R., Betts, A., Shapiro, J. W. & Marx, C. J. Adding biotic complexity alters the metabolic benefits of mutualism.
10. Mizuno, K. *et al.* Adjacent-possible ecological niche: growth of Lactobacillus species co-cultured with Escherichia coli in a synthetic minimal medium. *Sci Rep* **7**, 12880 (2017).
11. Noto Guillen, M., Rosener, B., Sayin, S. & Mitchell, A. Assembling stable syntrophic Escherichia coli communities by comprehensively identifying beneficiaries of secreted goods. *Cell Systems* **12**, 1064-1078.e7 (2021).
12. Yu, H. Bacterial chemolithoautotrophy via manganese oxidation.
13. Xu, X. *et al.* Bacterial growth and environmental adaptation via thiamine biosynthesis and thiamine-mediated metabolic interactions. *The ISME Journal* **18**, wrae157 (2024).
14. Bisesi, A. T. *et al.* Bacteriophage specificity is impacted by interactions between bacteria. *mSystems* **9**, e01177-23 (2024).

15. Pherribo, G. J. & Taga, M. E. Bacteriophage-mediated lysis supports robust growth of amino acid auxotrophs. *The ISME Journal* **17**, 1785–1788 (2023).
16. Chodkowski, J. L. & Shade, A. Bioactive exometabolites drive maintenance competition in simple bacterial communities. *mSystems* **9**, e00064-24 (2024).
17. Lee, K. W. K. *et al.* Biofilm development and enhanced stress resistance of a model, mixed-species community biofilm. *The ISME Journal* **8**, 894–907 (2014).
18. Castledine, M. *et al.* Characterizing a stable five-species microbial community for use in experimental evolution and ecology. *Microbiology* **170**, (2024).
19. Meroz, N., Tovi, N., Sorokin, Y. & Friedman, J. Community composition of microbial microcosms follows simple assembly rules at evolutionary timescales. *Nat Commun* **12**, 2891 (2021).
20. Pearl Mizrahi, S., Goyal, A. & Gore, J. Community interactions drive the evolution of antibiotic tolerance in bacteria. *Proc. Natl. Acad. Sci. U.S.A.* **120**, e2209043119 (2023).
21. Friedman, J. Community structure follows simple assembly rules in microbial microcosms.
22. Zuroff, T. R., Xiques, S. B. & Curtis, W. R. Consortia-mediated bioprocessing of cellulose to ethanol with a symbiotic Clostridium phytofermentans/yeast co-culture. *Biotechnol Biofuels* **6**, 59 (2013).
23. Kang, D. *et al.* Construction of Simplified Microbial Consortia to Degrade Recalcitrant Materials Based on Enrichment and Dilution-to-Extinction Cultures. *Front. Microbiol.* **10**, 3010 (2020).
24. Bittleston, L. S. Context-dependent dynamics lead to the assembly of functionally distinct microbial communities.
25. Lopes, W., Amor, D. R. & Gore, J. Cooperative growth in microbial communities is a driver of multistability. *Nat Commun* **15**, 4709 (2024).
26. Pacheco, A. R. Costless metabolic secretions as drivers of interspecies interactions in microbial ecosystems.
27. Hong, Y.-J., Cai, Y. & Antoniewicz, M. R. Cross-feeding of amino acid pathway intermediates is common in co-cultures of auxotrophic Escherichia coli. *Metabolic Engineering* **88**, 172–179 (2025).

28. Mueller, A. J., Daebeler, A., Herbold, C. W., Kirkegaard, R. H. & Daims, H. Cultivation and genomic characterization of novel and ubiquitous marine nitrite-oxidizing bacteria from the *Nitrospirales*. *The ISME Journal* **17**, 2123–2133 (2023).
29. Lim, Y., Seo, J.-H., Giovannoni, S. J., Kang, I. & Cho, J.-C. Cultivation of marine bacteria of the SAR202 clade. *Nat Commun* **14**, 5098 (2023).
30. Shade, A. *et al.* Culturing captures members of the soil rare biosphere. *Environmental Microbiology* **14**, 2247–2252 (2012).
31. Summers, Z. M. *et al.* Direct Exchange of Electrons Within Aggregates of an Evolved Syntrophic Coculture of Anaerobic Bacteria. *Science* **330**, 1413–1415 (2010).
32. Grant, M. A. A., Kazamia, E., Cicuta, P. & Smith, A. G. Direct exchange of vitamin B12 is demonstrated by modelling the growth dynamics of algal–bacterial cocultures. *The ISME Journal* **8**, 1418–1427 (2014).
33. Zhou, K., Qiao, K., Edgar, S. & Stephanopoulos, G. Distributing a metabolic pathway among a microbial consortium enhances production of natural products. *Nat Biotechnol* **33**, 377–383 (2015).
34. Reyes-González, D. *et al.* Dynamic proteome allocation regulates the profile of interaction of auxotrophic bacterial consortia. *R. Soc. open sci.* **9**, 212008 (2022).
35. Chang, C.-Y. & Baji, D. Emergent coexistence in multispecies microbial communities. (2023).
36. Wintermute, E. H. & Silver, P. A. Emergent cooperation in microbial metabolism. *Molecular Systems Biology* **6**, 407 (2010).
37. Goldford, J. E. *et al.* Emergent simplicity in microbial community assembly. (2018).
38. Bao, T., Qian, Y., Xin, Y., Collins, J. J. & Lu, T. Engineering microbial division of labor for plastic upcycling. *Nat Commun* **14**, 5712 (2023).
39. Gopalakrishnappa, C., Li, Z. & Kuehn, S. Environmental modulators of algae-bacteria interactions at scale.
40. Hillesland, K. L. *et al.* Erosion of functional independence early in the evolution of a microbial mutualism. *Proc. Natl. Acad. Sci. U.S.A.* **111**, 14822–14827 (2014).
41. Urui, M. *et al.* Establishment of a co-culture system using Escherichia coli and Pichia pastoris (Komagataella phaffii) for valuable alkaloid production. *Microb Cell Fact* **20**, 200 (2021).

42. Cao, L., Garcia, S. L. & Wurzbacher, C. Establishment of microbial model communities capable of removing trace organic chemicals for biotransformation mechanisms research. *Microb Cell Fact* **22**, 245 (2023).
43. Benninghaus, L., Schwardmann, L. S., Jilg, T. & Wendisch, V. F. Establishment of synthetic microbial consortia with *Corynebacterium glutamicum* and *Pseudomonas putida* : Design, construction, and application to production of γ-glutamylisopropylamide and l -theanine. *Microbial Biotechnology* **17**, e14400 (2024).
44. Jones, J. A. Experimental and computational optimization of an Escherichia coli co-culture for the efficient production of flavonoids. *Metabolic Engineering* (2016).
45. Liu, H. *et al.* FACS-iChip: a high-efficiency iChip system for microbial 'dark matter' mining. *Mar Life Sci Technol* **3**, 162–168 (2021).
46. Pande, S. Fitness and stability of obligate cross-feeding interactions that emerge upon gene loss in bacteria. *The ISME Journal*.
47. Wang, J. *et al.* Formation of a constructed microbial community in a nutrient-rich environment indicates bacterial interspecific competition. *mSystems* **9**, e00006-24 (2024).
48. Müller, M. J. I., Neugeboren, B. I., Nelson, D. R. & Murray, A. W. Genetic drift opposes mutualism during spatial population expansion. *Proc. Natl. Acad. Sci. U.S.A.* **111**, 1037–1042 (2014).
49. Ganesan, V. Heterologous biosynthesis of natural product naringenin by co-culture engineering. *Synthetic and Systems Biotechnology* (2017).
50. Ren, D., Madsen, J. S., Sørensen, S. J. & Burmølle, M. High prevalence of biofilm synergy among bacterial soil isolates in cocultures indicates bacterial interspecific cooperation. *The ISME Journal* **9**, 81–89 (2015).
51. Font-Verdera, F., Liébana, R., Rossello-Mora, R. & Viver, T. Impact of dilution on stochastically driven methanogenic microbial communities of hypersaline anoxic sediments. *FEMS Microbiology Ecology* **99**, fiad146 (2023).
52. Dooley, K. D., Henry, L. P. & Bergelson, J. Impact of timing on the invasion of synthetic bacterial communities. *The ISME Journal* **18**, wrae220 (2024).
53. Weiss, A. S. *et al.* In vitro interaction network of a synthetic gut bacterial community. *The ISME Journal* **16**, 1095–1109 (2022).

54. Bartelme, R. P. *et al.* Influence of Substrate Concentration on the Culturability of Heterotrophic Soil Microbes Isolated by High-Throughput Dilution-to-Extinction Cultivation. **5**, (2020).
55. Yu, X. Interactions in self-assembled microbial communities saturate with diversity.
56. Aziz, F. A. A. *et al.* Interspecies interactions are an integral determinant of microbial community dynamics. *Front. Microbiol.* **6**, (2015).
57. Strassmann, J. E. Joan E. Strassmann. *Current Biology* **18**, R986–R988 (2008).
58. Wienhausen, G. *et al.* Ligand cross-feeding resolves bacterial vitamin B12 auxotrophies. *Nature* **629**, 886–892 (2024).
59. Hammarlund, S. P., Gedeon, T., Carlson, R. P. & Harcombe, W. R. Limitation by a shared mutualist promotes coexistence of multiple competing partners. *Nat Commun* **12**, 619 (2021).
60. Yu, X. A. *et al.* Low-level resource partitioning supports coexistence among functionally redundant bacteria during successional dynamics. *The ISME Journal* **18**, wrad013 (2024).
61. Giri, S. *et al.* Metabolic dissimilarity determines the establishment of cross-feeding interactions in bacteria. *Current Biology* **31**, 5547-5557.e6 (2021).
62. Harcombe, W. R. *et al.* Metabolic Resource Allocation in Individual Microbes Determines Ecosystem Interactions and Spatial Dynamics. *Cell Reports* **7**, 1104–1115 (2014).
63. Liu, Z. *et al.* Metabolite Cross-Feeding between Rhodococcus ruber YYL and Bacillus cereus MLY1 in the Biodegradation of Tetrahydrofuran under pH Stress. *Applied and Environmental Microbiology* **85**, (2019).
64. Bayer, B. *et al.* Metabolite release by nitrifiers facilitates metabolic interactions in the ocean. *The ISME Journal* **18**, wrae172 (2024).
65. Belzer, C. *et al.* Microbial Metabolic Networks at the Mucus Layer Lead to Diet-Independent Butyrate and Vitamin B12 Production by Intestinal Symbionts. **8**, (2017).
66. LaSarre, B., McCully, A. L., Lennon, J. T. & McKinlay, J. B. Microbial mutualism dynamics governed by dose-dependent toxicity of cross-fed nutrients. *The ISME Journal* **11**, 337–348 (2017).
67. Micali, G., Hockenberry, A. M., Dal Co, A. & Ackermann, M. Minorities drive growth resumption in cross-feeding microbial communities. *Proc. Natl. Acad. Sci. U.S.A.* **120**, e2301398120 (2023).

68. Garcia, S. L. *et al.* Model Communities Hint at Promiscuous Metabolic Linkages between Ubiquitous Free-Living Freshwater Bacteria. **3**, (2018).
69. Melero-Jiménez, I. J. *et al.* Mutualism breakdown underpins evolutionary rescue in an obligate cross-feeding bacterial consortium. *Nat Commun* **16**, 3482 (2025).
70. Hom, E. F. Y. & Murray, A. W. Niche engineering demonstrates a latent capacity for fungal-algal mutualism. *Science* **345**, 94–98 (2014).
71. Pacheco, A. R., Osborne, M. L. & Segrè, D. Non-additive microbial community responses to environmental complexity. *Nat Commun* **12**, 2365 (2021).
72. Harcombe, W. NOVEL COOPERATION EXPERIMENTALLY EVOLVED BETWEEN SPECIES. *Evolution* https://doi.org/10.1111/j.1558-5646.2010.00959.x (2010) doi:10.1111/j.1558-5646.2010.00959.x.
73. Estrela, S., Sanchez-Gorostiaga, A., Vila, J. C. & Sanchez, A. Nutrient dominance governs the assembly of microbial communities in mixed nutrient environments. *eLife* **10**, e65948 (2021).
74. Qiao, Y. *et al.* Nutrient status changes bacterial interactions in a synthetic community. *Appl Environ Microbiol* **90**, e01566-23 (2024).
75. Oña, L. *et al.* Obligate cross-feeding expands the metabolic niche of bacteria. *Nat Ecol Evol* **5**, 1224–1232 (2021).
76. Di Martino, R., Picot, A. & Mitri, S. Oxidative stress changes interactions between 2 bacterial species from competitive to facilitative. *PLoS Biol* **22**, e3002482 (2024).
77. Marchand, N. & Collins, C. H. Peptide-based communication system enables *Escherichia coli* to *Bacillus megaterium* interspecies signaling. *Biotech & Bioengineering* **110**, 3003–3012 (2013).
78. Rodríguez-Verdugo, A. & Ackermann, M. Rapid evolution destabilizes species interactions in a fluctuating environment. *The ISME Journal* **15**, 450–460 (2021).
79. Hillesland, K. L. & Stahl, D. A. Rapid evolution of stability and productivity at the origin of a microbial mutualism. *Proc. Natl. Acad. Sci. U.S.A.* **107**, 2124–2129 (2010).
80. Aulakh, S. K. *et al.* Spontaneously established syntrophic yeast communities improve bioproduction. *Nat Chem Biol* **19**, 951–961 (2023).
81. Ratzke, C. Strength of species interactions determines biodiversity and stability in microbial communities. **4**, (2020).

82. Shou, W., Ram, S. & Vilar, J. M. G. Synthetic cooperation in engineered yeast populations. *Proc. Natl. Acad. Sci. U.S.A.* **104**, 1877–1882 (2007).
83. Zuñiga, C. *et al.* Synthetic microbial communities of heterotrophs and phototrophs facilitate sustainable growth. *Nat Commun* **11**, 3803 (2020).
84. Mee, M. T., Collins, J. J., Church, G. M. & Wang, H. H. Syntrophic exchange in synthetic microbial communities. *Proc. Natl. Acad. Sci. U.S.A.* **111**, (2014).
85. Gao, C.-H., Cao, H., Cai, P. & Sørensen, S. J. The initial inoculation ratio regulates bacterial coculture interactions and metabolic capacity. *The ISME Journal* **15**, 29–40 (2021).
86. Ho, A. *et al.* The more, the merrier: heterotroph richness stimulates methanotrophic activity. *The ISME Journal* **8**, 1945–1948 (2014).
87. Mao, Z. *et al.* The selection of copiotrophs may complicate biodiversity-ecosystem functioning relationships in microbial dilution-to-extinction experiments. *Environmental Microbiome* **18**, 19 (2023).
88. Muzafar, S., Nair, R. R., Andersson, D. I. & Warsi, O. M. The strength of interspecies interaction in a microbial community determines its susceptibility to invasion. *PLoS Biol* **22**, e3002889 (2024).
89. Sanz-Sáez, I. *et al.* Top abundant deep ocean heterotrophic bacteria can be retrieved by cultivation. *ISME Communications* **3**, 92 (2023).
90. Bohannan, B. J. M., Kerr, B., Jessup, C. M. & Hughes, J. B. Trade-offs and coexistence in microbial microcosms.
91. Yang, J. W. *et al.* Trade-Offs between Competitive Ability and Resistance to Top-Down Control in Marine Microbes. *mSystems* **8**, e01017-22 (2023).
92. Gabrielli, N. *et al.* Unravelling metabolic cross-feeding in a yeast–bacteria community using ^{13}C -based proteomics. *Molecular Systems Biology* **19**, e11501 (2023).
