# Supplementary material for: Microbial model communities exhibit widespread metabolic interdependencies
Source: Commun Biol. 2025 Dec 3;8:1752. doi: 10.1038/s42003-025-09306-y (PMC12680743; doi:10.1038/s42003-025-09306-y)
Supplement: Supplementary file 2 — Description of Additional Supplementary Materials [file 42003_2025_9306_MOESM2_ESM.pdf]

Description of Additional Supplementary Files

File name: Supplementary Data 1

Description: Overview of culture studies investigating microbial interactions.

File name: Supplementary Data 2

Description: Metadata of environmental metagenomic samples used in this study (n = 112), including their accession number and reference.

File name: Supplementary Data 3

Description: List of sequenced microbial model communities and their accession numbers in NCBI.

File name: Supplementary Data 4

Description: Metadata of all 1603 medium-high quality MAGs (>45% completeness and <10% contamination) binned in this study. For each MAG, we included genomic statistics such as completeness, contamination, GC, estimated genome size, number of contigs, N50, coding density, number of predicted genes, GTDB-tk taxonomy, general origin (culture or environment), and species cluster representative. For MAGs coming from cultures, we included characteristics such as inoculum size, and MDA.

File name: Supplementary Data 5

Description: Relative abundance results of the 701 species-clusters representative across the 112 Baltic Sea metagenomes.

File name: Supplementary Data 6

Description: Pathway completeness of the selected default KEGG modules involved in the biosynthesis of amino acids and B vitamins for each 701 species-cluster.

File name: Supplementary Data 7

Description: Overview of the 30 custom KEGG biosynthetic modules used in this study, including amino acid/vitamin name, precursor, module combinations, 3-letter code, and module ID.

File name: Supplementary Data 8

Description: Amino acid custom module information and definition rules based on defualt KEGG modules or KEGG Pathway Maps.

File name: Supplementary Data 9

Description: B vitamin custom module information and definition rules based on defualt KEGG modules or KEGG Pathway Maps.

File name: Supplementary Data 10

Description: Pathway completeness of the selected default KEGG modules involved in the biosynthesis of amino acids and B vitamins for each 701 species-cluster.

File name: Supplementary Data 11

Description: Pathway completeness of the custom KEGG modules for the biosynthesis of amino acids and B vitamins for each high-quality cultivated genome (n = 305). The last column indicates the community complexity category (1, 2, 3, or >3 genomes per culture).

File name: Supplementary Data 12

Description: Source data for Figure 9, including species biosynthetic completeness group (low, medium, high), UMAP-based subgroup, cultivated species cluster assignment, phylum, estimated genome size, mean relative abundance, and mean completeness of amino acid and vitamin modules.
