# Supplementary material for: Microbial model communities exhibit widespread metabolic interdependencies
Source: Commun Biol. 2025 Dec 3;8:1752. doi: 10.1038/s42003-025-09306-y (PMC12680743; doi:10.1038/s42003-025-09306-y)
Supplement: Supplementary file 4 — Reporting Summary [file 42003_2025_9306_MOESM4_ESM.pdf]

nature portfolio

Corresponding author(s): Armando Pacheco-Valenciana
Sarahi L. Garcia

Last updated by author(s): Aug 15, 2025

Reporting Summary

Nature Portfolio wishes to improve the reproducibility of the work that we publish. This form provides structure for consistency and transparency in reporting. For further information on Nature Portfolio policies, see our Editorial Policies and the Editorial Policy Checklist.

Statistics

For all statistical analyses, confirm that the following items are present in the figure legend, table legend, main text, or Methods section.

n/a	Confirmed	
☐	☒	The exact sample size (*n*) for each experimental group/condition, given as a discrete number and unit of measurement
☒	☐	A statement on whether measurements were taken from distinct samples or whether the same sample was measured repeatedly
☐	☒	The statistical test(s) used AND whether they are one- or two-sided<br>*Only common tests should be described solely by name; describe more complex techniques in the Methods section.*
☒	☐	A description of all covariates tested
☐	☒	A description of any assumptions or corrections, such as tests of normality and adjustment for multiple comparisons
☐	☒	A full description of the statistical parameters including central tendency (e.g. means) or other basic estimates (e.g. regression coefficient) AND variation (e.g. standard deviation) or associated estimates of uncertainty (e.g. confidence intervals)
☒	☐	For null hypothesis testing, the test statistic (e.g. *F*, *t*, *r*) with confidence intervals, effect sizes, degrees of freedom and *P* value noted<br>*Give P values as exact values whenever suitable.*
☒	☐	For Bayesian analysis, information on the choice of priors and Markov chain Monte Carlo settings
☒	☐	For hierarchical and complex designs, identification of the appropriate level for tests and full reporting of outcomes
☒	☐	Estimates of effect sizes (e.g. Cohen's *d*, Pearson's *r*), indicating how they were calculated

Our web collection on statistics for biologists contains articles on many of the points above.

Software and code

Policy information about availability of computer code

Data collection	No software was used to collect data
Data analysis	Trimmomatic v0.36, MetaWRAP v1.3.2, QUAST v5.0.2, Bowtie2, samtool v1.9, metaBAT2, maxBIN2, CONOCT, GTDB-tk v2.1.1, CheckM v1.1.3, Stobrealign V0.14.0, Anvi'o V7.1, R v4.4.0

For manuscripts utilizing custom algorithms or software that are central to the research but not yet described in published literature, software must be made available to editors and reviewers. We strongly encourage code deposition in a community repository (e.g. GitHub). See the Nature Portfolio guidelines for submitting code & software for further information.

Data

Policy information about availability of data

All manuscripts must include a data availability statement. This statement should provide the following information, where applicable:

- Accession codes, unique identifiers, or web links for publicly available datasets
- A description of any restrictions on data availability
- For clinical datasets or third party data, please ensure that the statement adheres to our policy

Data has been deposited in NCBI under the BioProject ID PRJNA1134408

Research involving human participants, their data, or biological material

Policy information about studies with human participants or human data. See also policy information about sex, gender (identity/presentation), and sexual orientation and race, ethnicity and racism.

Reporting on sex and gender	n/a
Reporting on race, ethnicity, or other socially relevant groupings	n/a
Population characteristics	n/a
Recruitment	n/a
Ethics oversight	n/a

Note that full information on the approval of the study protocol must also be provided in the manuscript.

Field-specific reporting

Please select the one below that is the best fit for your research. If you are not sure, read the appropriate sections before making your selection.

☐ Life sciences ☐ Behavioural & social sciences ☒ Ecological, evolutionary & environmental sciences

For a reference copy of the document with all sections, see nature.com/documents/nr-reporting-summary-flat.pdf

Ecological, evolutionary & environmental sciences study design

All studies must disclose on these points even when the disclosure is negative.

Study description	We used the dilution-to-extinction technique to achieve approximately the desired starting number of cells in our cultures (aka microbial model communities). The cultures were designed in two ways: low inoculum size (LS) and high inoculum size (HS). The LS cultures included hundreds of individual cultures in 96-well plates with a starting number of cells of 0 (control), 2, 6, 10, 15, 20, 30, 50, and 100 cells/well. The HS cultures were prepared in bottles with starting numbers of cells of 0 (control), 200, 600, 1000, 1500, 2000, 3000, 5000, 10000, 100000, and 1000000 cells/bottle. All cultures were incubated with light/dark cycles (light 6:42, dark 18:32 each day) and 12.2 °C at light and 11.8 °C at dark for four weeks before further processing
Research sample	A water sample was taken from the Baltic Sea close to Askö in the Trosa Archipielago (Lat 58°48.20'N, Lon 17°37.42'E) in October 2021.
Sampling strategy	The sample was taken from a boat and in a canister and transported to the lab where it underwent further processing.
Data collection	n/a
Timing and spatial scale	n/a
Data exclusions	n/a
Reproducibility	In total 204 model communities produced MDA amplification that lead to metagenomic sequencing that lead to assembly and binning of metagenome-assembled genomes (MAGs). While this model communities are not biological replicates, they can act as ecological replicates when grouped according to the the number of MAGs found in each of them.
Randomization	We performed dilution cultivation which in itself randomizes which cell start each experiment together
Blinding	Data was analyzed in its entirety without a pre expected outcome

Did the study involve field work? ☐ Yes ☒ No

Reporting for specific materials, systems and methods

We require information from authors about some types of materials, experimental systems and methods used in many studies. Here, indicate whether each material, system or method listed is relevant to your study. If you are not sure if a list item applies to your research, read the appropriate section before selecting a response.

Materials & experimental systems

n/a	Involved in the study
☒	☐ Antibodies
☒	☐ Eukaryotic cell lines
☒	☐ Palaeontology and archaeology
☒	☐ Animals and other organisms
☒	☐ Clinical data
☒	☐ Dual use research of concern
☒	☐ Plants

Methods

n/a	Involved in the study
☒	☐ ChIP-seq
☒	☐ Flow cytometry
☒	☐ MRI-based neuroimaging

Plants

Seed stocks	n/a
Novel plant genotypes	n/a
Authentication	n/a
